# Molecular Evolution of the Protease Region in Norovirus Genogroup II

**DOI:** 10.3389/fmicb.2019.02991

**Published:** 2020-01-14

**Authors:** Keita Ozaki, Yuki Matsushima, Koo Nagasawa, Jumpei Aso, Takeshi Saraya, Keisuke Yoshihara, Koichi Murakami, Takumi Motoya, Akihide Ryo, Makoto Kuroda, Kazuhiko Katayama, Hirokazu Kimura

**Affiliations:** ^1^Graduate School of Health Sciences, Gunma Paz University, Takasaki, Japan; ^2^Niitaka Co., Ltd., Osaka, Japan; ^3^Division of Virology, Kawasaki City Institute for Public Health, Kawasaki, Japan; ^4^Eastern Chiba Medical Center, Togane, Japan; ^5^Department of Respiratory Medicine, Kyorin University School of Medicine, Mitaka, Japan; ^6^Department of Pediatric Infectious Diseases, Institute of Tropical Medicine, Nagasaki University, Nagasaki, Japan; ^7^Infectious Disease Surveillance Center, National Institute of Infectious Diseases, Musashimurayama, Japan; ^8^Ibaraki Prefectural Institute of Public Health, Mito, Japan; ^9^Department of Microbiology, Yokohama City University School of Medicine, Yokohama, Japan; ^10^Pathogen Genomics Center, National Institute of Infectious Diseases, Shinjuku, Japan; ^11^Laboratory of Viral Infection I, Graduate School of Infection Control Sciences, Kitasato Institute for Life Sciences, Kitasato University, Minato, Japan

**Keywords:** molecular evolution, norovirus, GII, bioinformatics, protease, negative selection

## Abstract

Noroviruses are a major cause of viral epidemic gastroenteritis in humans worldwide. The protease (Pro) encoded in open reading frame 1 (ORF1) is an essential enzyme for proteolysis of the viral polyprotein. Although there are some reports regarding the evolutionary analysis of norovirus GII-encoding genes, there are few reports focused on the *Pro* region. We analyzed the molecular evolution of the *Pro* region of norovirus GII using bioinformatics approaches. A time-scaled phylogenetic tree of the *Pro* region constructed using a Bayesian Markov chain Monte Carlo method indicated that the common ancestor of GII diverged from GIV around 1680 CE [95% highest posterior density (HPD), 1607–1749]. The GII *Pro* region emerged around 1752 CE (95%HPD, 1707–1794), forming three further lineages. The evolutionary rate of GII *Pro* region was estimated at more than 10^–3^ substitutions/site/year. The distribution of the phylogenetic distances of each genotype differed, and showed genetic diversity. Mapping of the negative selection and substitution sites of the Pro structure showed that the substitution sites in the Pro protein were mostly produced under neutral selection in positions structurally adjacent to the active sites for proteolysis, whereas negative selection was observed in residues distant from the active sites. The phylodynamics of GII.P4, GII.P7, GII.P16, GII.P21, and GII.P31 indicated that their effective population sizes increased during the period from 2005 to 2016 and the increase in population size was almost consistent with the collection year of these genotypes. These results suggest that the *Pro* region of the norovirus GII evolved rapidly, but under no positive selection, with a high genetic divergence, similar to that of the RNA-dependent RNA polymerase (*RdRp*) region and the *VP1* region of noroviruses.

## Introduction

Noroviruses (NoVs) are a major cause of acute epidemic viral gastroenteritis worldwide (Green, 2013; Robilotti et al., 2015). In developing countries, 200,000 deaths in children less than 5 years of age are estimated to be caused by NoV infections (Patel et al., 2008). Large-scale foodborne illnesses have been caused worldwide by NoVs (Le Guyader et al., 2008; Räsänen et al., 2010; Hardstaff et al., 2018; Sakon et al., 2018). It is estimated that NoVs are responsible for 699 million gastroenteritis cases per year, costing approximately $60 billion in medical and socioeconomic costs (Bartsch et al., 2016). Thus, NoV infection is a serious disease burden in many countries (Lopman et al., 2003; Kroneman et al., 2008; Hall et al., 2013).

NoV belongs to the family *Caliciviridae*, genus *Norovirus*, and is classified into 10 genogroups (GI-GX) (Chhabra et al., 2019). The GI, GII, GIV, GVIII and GIX genogroups of NoV can infect humans (Chhabra et al., 2019). GI and GII viruses are frequently detected in humans, and are further classified into 9 and 27 genotypes, respectively, based on the capsid region sequences (Chhabra et al., 2019). Previous molecular epidemiological studies have shown that some NoV GII genotypes, including GII.2, GII.3, GII.4, GII.6, and GII.17 are prevalent in different countries (Matsushima et al., 2015; Bruggink et al., 2016, 2018; Cannon et al., 2017; Wangchuk et al., 2017).

The NoV genome is a positive-sense, single-stranded RNA composed of three open reading frames (ORFs). ORF1 encodes six non-structural proteins including protease (Pro) and RNA-dependent RNA polymerase (RdRp). Pro plays a critical role in proteolytic cleavage of a large polyprotein encoded in the ORF1 (Liu et al., 1996; Blakeney et al., 2003). Thus, Pro is one of the essential enzymes in NoV propagation in host cells. Pro could be a major target for antiviral drugs, in addition to RdRp. Many studies have been conducted into the development of drug candidates (Hussey et al., 2011; Muhaxhiri et al., 2013; Muzzarelli et al., 2019; Viskovska et al., 2019). A recent report demonstrated different effectiveness of antivirals against GI and GII NoVs, suggesting that the amino acid diversity in Pro may result in different effectiveness of drugs (Viskovska et al., 2019). It is important to understand the evolution of the *Pro* region of GII NoV, because this virus is the predominant genogroup in patients with NoV infection. However, to the best of our knowledge, there are no reports related to a comprehensive molecular evolutionary analysis of the GII *Pro* region. We conducted a detailed evolutionary analysis of the NoV GII *Pro* region using large numbers of strains, and using the latest bioinformatics approaches.

## Materials and Methods

### Strain Selection

Full-length nucleotide sequences (543 nt) of the NoV GII *Pro* region were collected from GenBank^1^ (accessed on 17 November 2018). We classified these strains according to ORF1 using a norovirus genotyping tool (Kroneman et al., 2011) and selected all the sequences of the human NoV (HuNoV) GII. Strains with an unknown collection year and ambiguous sequences with undetermined nucleotides (such as N, Y, and V) were omitted from the dataset. After these eliminations, the dataset consisted of the *Pro* region sequences of approximately 1,500 strains. However, because of the limitations in the software’s capacity, it could not be used for the detection of recombination. Thus, we calculated the nucleotide identity among the 1,500 *Pro* region sequences using Clustal Omega (Sievers et al., 2011). We randomly selected one sequence from a group of homologous sequences with identity ≥99.8% and excluded the others from the dataset to reduce the sequences in the dataset. Furthermore, to estimate the recombination of the *Pro* region in the present strains, recombination analyses were performed using the RDP4.95 software with seven primary exploratory recombination signal detection methods: RDP, GENECONV, BOOTSCAN/RESCAN, MAXCHI, CHIMAERA, SISCAN, and 3SEQ (Martin et al., 2015). The threshold of the *p*-value for significance was set to 0.001. Recombinant regions were considered to be reliable when they were detected by more than four of these methods; however, no recombinant strains in the present sequences were estimated. Finally, a total of 760 strains were used in this study (Supplementary Table S1). The sequences in the dataset were aligned using the MAFFT software (Katoh and Standley, 2013).

### Construction of a Time-Scaled Phylogenetic Tree Using the Bayesian Markov Chain Monte Carlo Method

We constructed a time-scaled phylogenetic tree using the Bayesian MCMC method in the BEAST software package v2.4.8 (Drummond and Rambaut, 2007; Bouckaert et al., 2014). To estimate the phylogenetic relationships in the *Pro* region between distinct NoVs genogroups, we added the nucleotide sequences of human NoV GI (GI.P1), porcine GII (GII.P11 and GII.P18), bovine GIII (GIII.P1) and human GIV (GIV.P1) strains to the dataset, giving a total of 765 strains. We determined the best substitution model (GTR+I+Γ) using the jModelTest2 software (Guindon and Gascuel, 2003; Darriba et al., 2012). We then selected the best of four clock models – strict clock, relaxed clock exponential, relaxed clock log normal or random local clock – and two tree prior models, coalescent constant population and coalescent exponential population, using path sampling/stepping stone-sampling marginal-likelihood estimation (Baele et al., 2012). The dataset was analyzed using strict clock and tree prior of coalescent exponential population. The MCMC was run on chain lengths of 150,000,000 steps with sampling every 5,000 steps. The data were then evaluated for effective sample size using the Tracer^2^ software, and values greater than 200 were accepted. Maximum clade credibility trees were created by discarding the first 10% of the trees (burn-in) using TreeAnnotator v2.4.8 in the BEAST2 package. The time-scaled phylogenetic trees were visualized using FigTree^3^ v1.4.0 software. The reliability of branches was assessed using the 95% highest posterior density (HPD) interval. The evolutionary rates for the *Pro* region in the ORF1 genotypes of NoV GII including more than 10 strain sequences (P4, P7, P12, P16, P17, P21, and P31) were also estimated using the models determined from the datasets described above. We could not calculate the evolutionary rate for the *Pro* region of GII.P2 due to invalid data with a wide range of 95%HPD values.

### Calculation of Phylogenetic Distances

We created phylogenetic trees for the *Pro* region from the datasets of the NoV GII strains and each ORF1 genotype including more than 10 strains using the maximum likelihood (ML) method in the MEGA7 software package (Kumar et al., 2016). We determined the best substitution models using jModelTest2. We calculated the phylogenetic distances between NoV GII strains from the ML distance of the ML tree using the Patristic software (Fourment and Gibbs, 2006).

### Construction of Three-Dimensional Structures and Selective Pressure Analyses

We constructed structural models of the Pro proteins (GII.P1:U07611, GII.P2:DQ456824, GII.P3:KJ194500, GII.P4:AB 541272, GII.P5:KJ196288, GII.P6:AB039778, GII.P7:AB039777, GII.P8:AB039780, GII.P12:AB220922, GII.P16:KJ196286, GII. P17:AB983218, GII.P20:EU424333, GII.P21:KJ196284, GII.P24: MG495081, GII.P25:MG495083, GII.P30:AY134748, GII.P31: JX459907, GII.P32:MF405169, GII.P33:GQ845370, GII.P35:KC 576911, GII.P37:KJ194507, GII.P39:FJ537134, GII.P40:DQ3 66347, GII.P41:JX846924, GII.PNA5:MG495082, and GII.PNA7: MG557653) using the homology modeling software, MODELER v9.20 (Webb and Sali, 2014, 2016). The crystal structure of the GII.P4 Pro protein (PDB ID: 6NIR) was used as the template for homology modeling. Amino acid sequences of the template and targets were aligned using the MAFFTash software (Standley et al., 2007; Katoh et al., 2009). The constructed structures were minimized using GROMOS96 (van Gunsteren et al., 1996) implemented in Swiss PDB Viewer v4.1 (Guex and Peitsch, 1997), and structural reliability was evaluated using Ramachandran plots via the RAMPAGE server (Lovell et al., 2003). We identified favored regions of 97.49 ± 0.52%, allowed regions of 2.50 ± 0.51% and outlier regions of 0.01 ± 0.06% [mean ± standard deviation (SD)] of all residues in each structure. Non-synonymous (dN) and synonymous (dS) substitution rates at each codon were calculated to estimate the positive and negative selection sites in the NoV GII *Pro* regions and in the regions of each genotype including more than three strains using the Datamonkey server (Pond and Frost, 2005; Delport et al., 2010). We identified the consensus sites shown by three methods: single-likelihood ancestor counting (SLAC), fixed effects likelihood (FEL), and internal fixed effects likelihood (IFEL) using a significance level of *p* < 0.05. We assigned these consensus sites as sites under positive, negative or neutral selection. A two-tail extended binomial distribution was used to calculate the *p*-value for SLAC. The FEL and IFEL were based on a single degree of freedom likelihood ratio test using an asymptotic chi-squared distribution, in order to classify a site as positively or negatively selected. The final structural models were modified and colored using the Chimera v1.13 software (Pettersen et al., 2004). The substitution sites of the other Pro proteins were compared to a GII.P20 strain (accession no. EU424333) and negative selection at these sites was mapped onto the structure.

### Bayesian Skyline Plot Analysis

We estimated the genealogical population size of the *Pro* region in the ORF1 genotypes of NoV GII including >10 strain sequences (P2, P4, P7, P12, P16, P17, P21, and P31) using a Bayesian skyline plot algorithm using BEAST v2.4.8. Appropriate substitution and clock models were selected as described above. The plots were visualized with 95%HPD using Tracer^2^.

### Statistical Analyses

Statistical analyses were conducted using the EZR statistical software implementation of the Kruskal–Wallis test controlled for multiple comparisons using the Holm test for phylogenetic distances (Kanda, 2013). Detailed statistical data are presented in Supplementary Table S2.

## Results

### Time-Scaled Phylogenetic Tree Constructed Using the Bayesian MCMC Method

**FIGURE 1:**
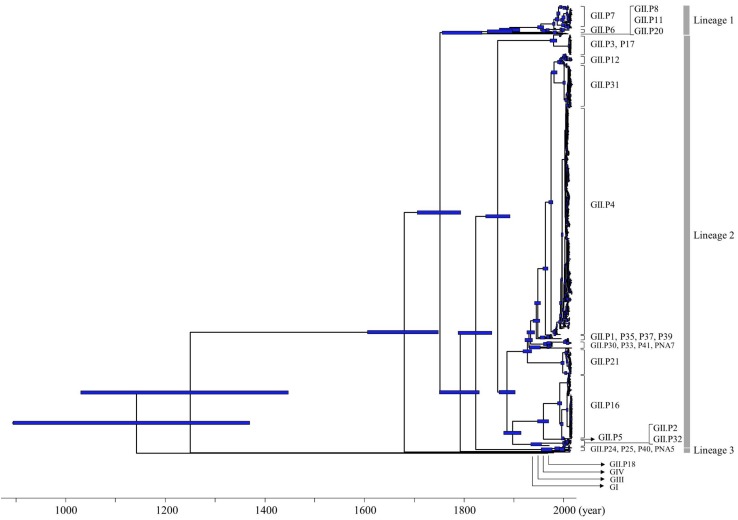
Time-scaled phylogenetic tree of full-length NoV *Pro* regions constructed using a Bayesian MCMC method. The maximum clade credibility tree of a dataset including the NoV GI, GII, GIII, and GIV genogroups is shown. Blue bars indicate the 95% HPD for each divergent year.

We constructed a time-scaled phylogenetic tree of the *Pro* region of NoV GII using a Bayesian MCMC method. The 26 ORF1 genotypes of the HuNoV GII strains were classified into three lineages by setting the cut-off value of phylogenetic distances for lineages as 0.9 substitutions/site (Figures 1, 3). With this threshold, we obtained lineage 1 (GII.P6-P8 and P20); lineage 2 (GII.P1-P5, P12, P16, P17, P21, P30–P33, P35, P37, P39, P41, and PNA7) and lineage 3 (GII.P24, P25, P40, and PNA5) (Figure 1). Strains with the GII.P4 formed many clusters of short duration.

The phylogenetic tree indicated that GI diverged from the common ancestor of GII, GIII, and GIV in 1143 CE (95%HPD, 895–1370), and GIII diverged from the common ancestor of GII and GIV in 1250 CE (95%HPD, 1031–1447). The common ancestor of GII diverged from GIV in 1680 CE (95%HPD, 1607–1749). The common ancestor of NoV GII viruses formed three lineages after 1752 CE (95%HPD, 1707–1794). Lineage 1 diverged in 1873 CE (95%HPD, 1847–1898), lineage 2 in 1868 CE (95%HPD, 1844–1892) and lineage 3 in 1966 CE (95%HPD, 1956–1977). We show the estimated divergence year for the three lineages, and the ORF1 genotypes including two or more strains in Table 1. GII.P20 diverged first from a common ancestor with the other HuNoV GII in 1873 CE (95%HPD, 1847–1898; Supplementary Figure S1).

**TABLE 1:** Year of divergence of each ORF1 genotype for HuNoV GII.

**Genogroup**	**Lineage**	**ORF1 genotypes**	**Year of divergence (95% HPD)**
GII	1	GII.P6-P8 and P20	1873 (1847–1898)
	2	GII.P1-P5, P12, P16, P17, P21, P30-P33, P35, P37, P39, P41, and PNA7	1868 (1844–1892)
	3	GII.P24, P25, P40, and PNA5	1966 (1956–1977)
		GII.P2	2001 (1999–2003)
		GII.P3	1993 (1992–1995)
		GII.P4	1981 (1979–1983)
		GII.P6	1961 (1956–1965)
		GII.P7	1981 (1978–1984)
		GII.P8	1983 (1979–1986)
		GII.P12	1992 (1988–1995)
		GII.P16	1992 (1988–1996)
		GII.P17	2011 (2010–2012)
		GII.P21	1998 (1995–2002)
		GII.P24	2012 (2011–2013)
		GII.P30	1970 (1967–1972)
		GII.P31	2001 (1999–2004)
		GII.P33	2003 (2000–2005)
		GII.P39	1969 (1967–1972)
		GII.P40	2000 (1998–2001)
		GII.P41	1964 (1960–1968)

*HPD, highest posterior density.*

**FIGURE 2:**
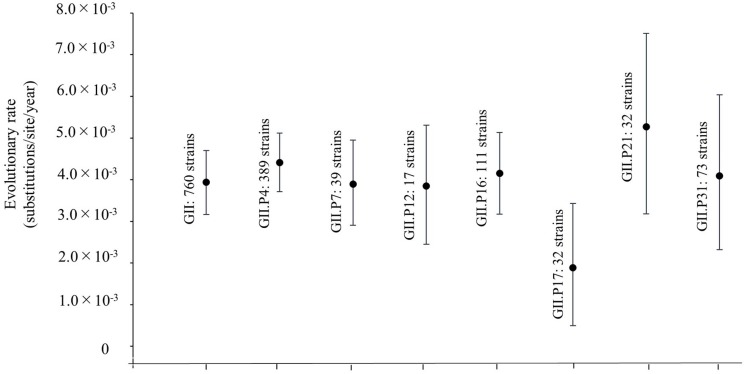
Evolutionary rates of the full-length nucleotide sequences of the NoV *Pro* regions. The *y-*axis represents the evolutionary rate (substitutions/site/year) and the *x*-axis indicates each genotype. The black circles indicate the mean and the bars indicate the interval of 95% HPD. The evolutionary rate of this region in overall HuNoV GII strains (760 strains) was estimated as 3.94 × 10^–3^ substitutions/site/year (95% HPD, 3.16–4.70 × 10^–3^ substitutions/site/year). The evolutionary rates of GII.P4 was 4.41 × 10^–3^ substitutions/site/year (95% HPD, 3.71–5.12 × 10^–3^ substitutions/site/year). The evolutionary rate of GII.P7 was 3.90 × 10^–3^ substitutions/site/year (95% HPD, 2.91–4.95 × 10^–3^ substitutions/site/year). The evolutionary rate of GII.P12 was 3.85 × 10^–3^ substitutions/site/year (95% HPD, 2.45–5.31 × 10^–3^ substitutions/site/year). The evolutionary rate of GII.P16 was 4.15 × 10^–3^ substitutions/site/year (95% HPD, 3.17–5.14 × 10^–3^ substitutions/site/year. The evolutionary rate of GII.P17 was 1.89 × 10^–3^ substitutions/site/year (95% HPD, 4.99 × 10^–4^–3.43 × 10^–3^ substitutions/site/year). The evolutionary rate of GII.P21 was 5.27 × 10^–3^ substitutions/site/year (95% HPD, 3.18–7.51 × 10^–3^ substitutions/site/year). The evolutionary rate of GII.P31 was 4.09 × 10^–3^ substitutions/site/year (95% HPD, 2.32–6.04 × 10^–3^ substitutions/site/year).

We also estimated the evolutionary rates for the *Pro* region in HuNoV GII strains and each ORF1 genotype (Figure 2). The evolutionary rate of this region in the HuNoV GII strains (760 strains) was estimated as 3.94 × 10^–3^ substitutions/site/year (95% HPD, 3.16–4.70 × 10^–3^ substitutions/site/year). The evolutionary rate of GII.P4 was 4.41 × 10^–3^ substitutions/site/year (95% HPD, 3.71–5.12 × 10^–3^ substitutions/site/year). The evolutionary rate of GII.P7 was 3.90 × 10^–3^ substitutions/site/year (95% HPD, 2.91–4.95 × 10^–3^ substitutions/site/year). The evolutionary rate of GII.P12 was 3.85 × 10^–3^ substitutions/site/year (95% HPD, 2.45–5.31 × 10^–3^ substitutions/site/year). The evolutionary rate of GII.P16 was 4.15 × 10^–3^ substitutions/site/year (95% HPD, 3.17–5.14 × 10^–3^ substitutions/site/year. The evolutionary rate of GII.P17 was 1.89 × 10^–3^ substitutions/site/year (95% HPD, 4.99 × 10^–4^–3.43 × 10^–3^ substitutions/site/year). The evolutionary rate of GII.P21 was 5.27 × 10^–3^ substitutions/site/year (95% HPD, 3.18–7.51 × 10^–3^ substitutions/site/year). The evolutionary rate of GII.P31 was 4.09 × 10^–3^ substitutions/site/year (95% HPD, 2.32–6.04 × 10^–3^ substitutions/site/year). The average evolutionary rates, including 95%HPD ranges, showed no overlap between GII.P17 and GII.P4, which suggests different evolutionary rates.

### Phylogenetic Distances of the *Pro* Region in Norovirus GII Strains

**FIGURE 3:**
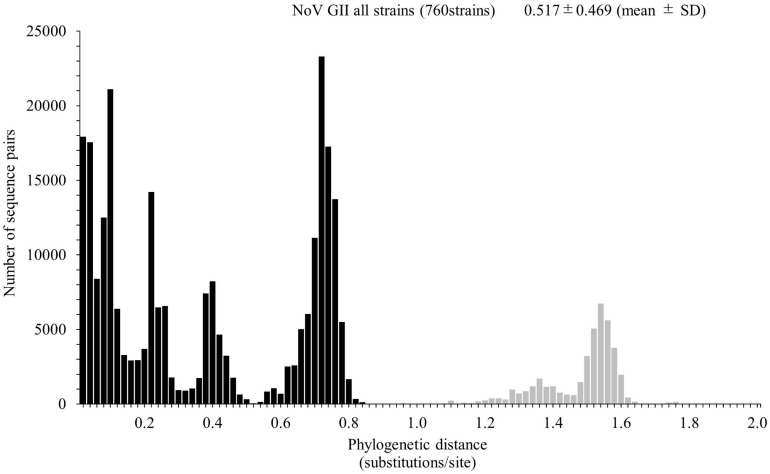
Distribution of phylogenetic distances of GII strains. The phylogenetic distance among NoV GII strains was calculated from the maximum likelihood (ML) tree constructed by the ML method. The *y*-axis represents the number of sequence pairs corresponding to each distance, and the *x*-axis shows the phylogenetic distance (substitutions/site). The distribution of phylogenetic distance for≥0.9 substitutions/site is shown in gray. The numbers on the histograms indicate the mean ± SD of the phylogenetic distance for GII strains.

**FIGURE 4:**
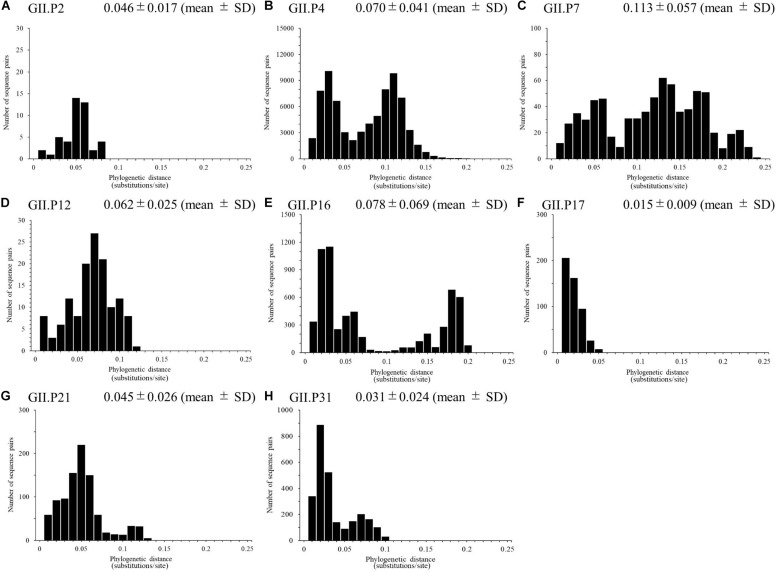
Phylogenetic distance between sequences of the full-length NoV *Pro* region. The phylogenetic distance between each genotype strain in NoV GII was calculated from the ML tree constructed by the ML method. The distributions of phylogenetic distances of NoV GII.P2 **(A)**, GII.P4 **(B)**, GII.P7 **(C)**, GII.P12 **(D)**, GII.P16 **(E)**, GII.P17 **(F)**, GII.P21 **(G)**, and GII.P31 **(H)** are shown. The *y*-axis represents the number of sequence pairs corresponding to each distance, and the *x*-axis shows the phylogenetic distance (substitutions/site). The numbers on the histograms indicate the mean ± SD of the phylogenetic distances for each genotype **(A–H)**. The statistical results for multiple comparisons are shown in Supplementary Table S2.

We calculated the number of nucleotide substitutions per site (phylogenetic distance) among the strains and showed the distribution of the distance to estimate the genetic diversity of the ORF1 genotypes and to compare the number between the genotypes in the *Pro* region of NoV GII. The average number of substitutions of overall NoV GII was 0.517 ± 0.469 per site in the region (mean ± SD; Figure 3). The phylogenetic distances of GII.P4 and GII.P7 were 0.070 ± 0.041 and 0.113 ± 0.057 substitutions/site, respectively. These histograms were distributed with a broad range of distances, indicating accumulating evolution with viral detection for long periods from the emergence of common ancestors (Figures 4B,C). The phylogenetic distances of GII.P2, GII.P12, GII.P17, GII.P21, and GII.P31 were 0.046 ± 0.017, 0.062 ± 0.025, 0.015 ± 0.009, 0.045 ± 0.026, and 0.031 ± 0.024 substitutions/site, respectively. These histograms were distributions with a narrow range of distances, suggesting shorter evolutionary process from the emergence of common ancestors than GII.P4 and GII.P7 (Figures 4A,D,F–H). The phylogenetic distance of GII.P16 was 0.078 ± 0.069 substitutions/site. This histogram showed a bimodal distribution, indicating the form of genetically distant two clusters (Figure 4E). The phylogenetic distances of each ORF1 genotype differed significantly among the ORF1 genotypes in NoV GII (*p* < 0.001), except for some ORF1 genotypes. Detailed data are presented in Supplementary Table S2.

### Mapping of Amino Acid Substitutions and Negative Selection Sites on the Structures of the Norovirus GII Pro Protein

**FIGURE 5:**
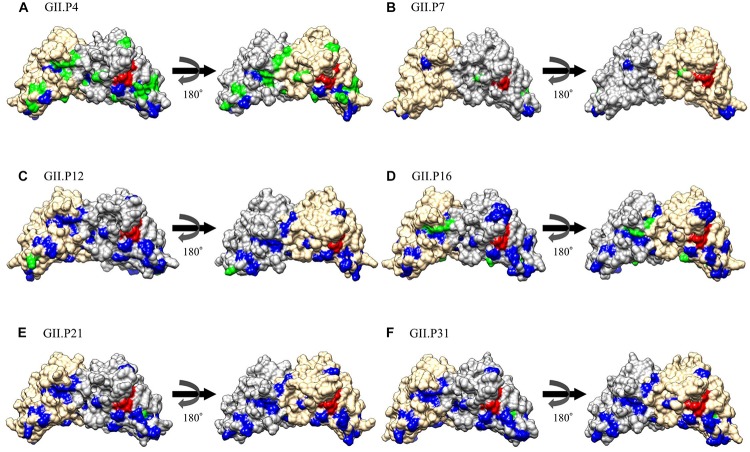
Structural models for the Pro protein of each genotype. Three-dimensional Pro dimer structures for GII.P4 **(A)**, GII.P7 **(B)**, GII.P12 **(C)**, GII.P16 **(D)**, GII.P21 **(E)**, and GII.P31 **(F)** are shown. The chains composing the dimer structures are colored gray (chain A) and Navajo white (chain B). Negative selection sites are colored green. Amino acid substitutions of the other genotypes compared to a GII.P20 strain are colored blue. Active site residues are colored red.

The substitution sites of the other Pro proteins to GII.P20 were mapped using GII.P20 (Accession no. EU424333) as a reference strain, because GII.P20 was the first to diverge from the common ancestor of the HuNoV GII *Pro* region (Supplementary Figure S1). We found no substitutions on the active site (H30, H54, and C139) of the protease among GII strains. However, some amino acid substitutions were found near the active site of GII Pro proteins. These substitutions were mostly neutral selection sites (Figure 5 and Supplementary Figure S2). We also mapped the negative selection sites and amino acid substitution sites on the Pro protein structures of NoV GII. Of the substitution sites in each ORF1 genotype, the GII.P4 and GII.P16 strains contained 20 and 6 negative selection sites per monomer, respectively (Table 2), and these sites were distant from the active site (Figure 5). There were five or fewer negative selection sites per monomer in some ORF1 genotypes (GII.P7, P12, P21, and P31), while no negative selection sites were found in the GII.P1, P2, P3, P6, P17, P30, P33, P39, P40, and P41 strains (Table 2). The consensus amino acid site under negative selection among three ORF1 genotypes was 58Leu (GII.P4, P21, and P31) (Supplementary Table S3). The consensus sites between two ORF1 genotypes were 11Ser (GII.P4 and P16), 25Phe (GII.P4 and P16), 47Ile (GII.P4 and P7), 62Lys (GII.P4 and P12), 87Ile (GII.P4 and P7), 129Ser and Gly (GII.P4 and P16), 135Thr and Gly (GII.P4 and P7), 151Tyr (GII.P4 and P16) (Supplementary Table S3). These sites under the negative selection were highly conserved, except for 129 and 135 amino acid positions. No positive selection sites were predicted in all strains of NoV GII genotypes (data not shown). The residues of amino acid under neutral selection were 12Phe and Leu, 14Ser and Thr, 25Phe and Leu, 26Ile and Val, 28Ser and Thr, 32Leu and Ile, 34Ala, Lys, Gln, Pro and Ser, 35Gly and Asn (Supplementary Table S3).

**TABLE 2:** The number of amino acid substitution sites and negative selection sites in the HuNoV GII strains.

**ORF1 genotypes**	**Number of substitution sites**	**Number of negative selection sites^‡^**
GII.P1	35	0 (0%)
GII.P2	38	0 (0%)
GII.P3	33	0 (0%)
GII.P4	36	20 (55.6%)
GII.P6	8	0 (0%)
GII.P7	7	3 (42.9%)
GII.P12	35	1 (2.9%)
GII.P16	35	6 (17.1%)
GII.P17	32	0 (0%)
GII.P21	35	1 (2.9%)
GII.P30	36	0 (0%)
GII.P31	36	1 (2.8%)
GII.P33	36	0 (0.0%)
GII.P39	35	0 (0.0%)
GII.P40	38	0 (0.0%)
GII.P41	35	0 (0.0%)

*^†^The number of substitution sites per monomer of other Pro proteins compared to the GII.P20 strain are presented. ^‡^The number of negative selection sites on the substitution sites. The negative selection sites were identified as the consensus sites shown by the SLAC, FEL, and IFEL methods.*

### Phylodynamics of the *Pro* Region in the Norovirus GII Strains Using the BSP Method

**FIGURE 6:**
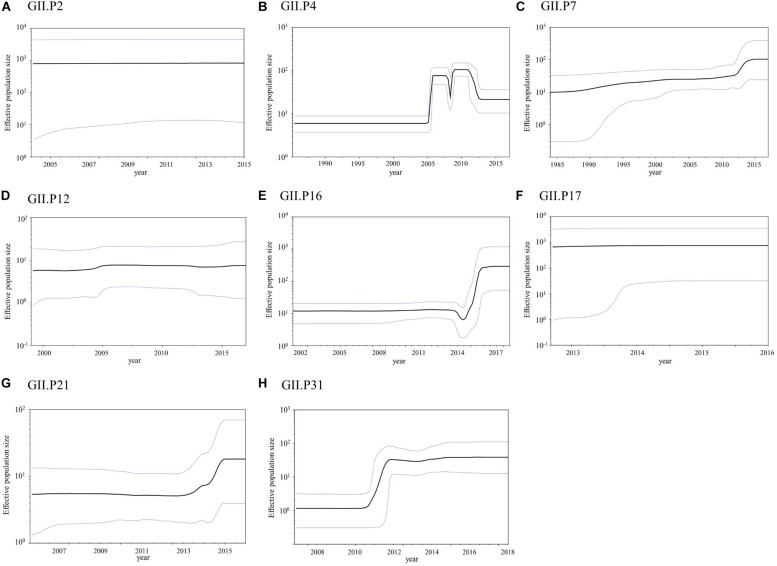
Bayesian skyline plot for the NoV GII strains. Plots for GII.P2 **(A)**, GII.P4 **(B)**, GII.P7 **(C)**, GII.P12 **(D)**, GII.P16 **(E)**, GII.P17 **(F)**, GII.P21 **(G)**, and GII.P31 **(H)** are shown. The *y*-axis represents the effective population size on a logarithmic scale, while the *x*-axis denotes the time in years. The solid black line indicates the mean posterior value and intervals with 95% HPD are represented by blue lines.

The phylodynamics of the NoV GII *Pro* region were analyzed using the BSP method and detailed parameters are shown in Supplementary Table S4. The mean effective population size of GII.P4 increased around 2005 and 2008 (Figure 6B). The mean effective population sizes of GII.P7, GII.P16, GII.P21, and GII.P31 increased around 2012, 2015–2016, 2014–2015, and 2011–2012, respectively, while no changes in the population size of the *Pro* region in other ORF1 genotypes were observed (Figures 6A,C–H).

## Discussion

We studied the molecular evolution of the *Pro* regions in NoV GII. To the best of our knowledge, there is no comprehensive molecular evolutionary study focused on the NoV *Pro* regions. Our findings suggest that (i) the common ancestor of the *Pro* region of the analyzed strains, including GI, GII, GIII, and GIV, diverged around 880 years ago (1143 CE), and the GII *Pro* region diverged from GIV around 340 years ago (1680 CE) and formed three major lineages of NoV GII strains around 270 years ago (1752 CE); (ii) the estimated evolutionary rate of the *Pro* region was >10^–3^ substitutions/site/year, and the values for each genotype were different; (iii) several amino acid substitutions (7–38 sites per monomer) in the GII protease were detected under neutral selection proximal to the active sites and under negative selection in the distant residues from the sites, and (iv) the phylodynamics of the GII *Pro* region showed different patterns of fluctuation between genotypes. These results suggested that the *Pro* region of the NoV GII evolved rapidly with amino acid substitutions under no positive selection, and the evolutionary trends were different between the ORF1 genotypes.

A time-scaled phylogenetic tree constructed using a Bayesian MCMC method indicated that the *Pro* region showed similar evolutionary process to the *RdRp* region, but was different from the *VP1* region at post emergence of the GII cluster, including the generations of three lineages (Kobayashi et al., 2016; Ozaki et al., 2018). We estimated that the *Pro* region, the *RdRp* region and the *VP1* region diverged around 270 years ago (1752 CE), around 290 years ago (1731 CE) and around 390 years ago (1630 CE), respectively, to form the GII cluster. The years of divergence of the *Pro* and *RdRp* regions were very similar (270 vs. 290 years ago), whereas the year of divergence of the *VP1* region was different by approximately 100 years. The year in divergence of each lineage was comparable between the *Pro* and *RdRp* regions (Ozaki et al., 2018). We estimated that in lineage 1 the *Pro* region diverged around 150 years ago (1873 CE) and the *RdRp* region around 170 years ago (1847 CE). In lineage 2, the *Pro* and the *RdRp* regions diverged around 150 years ago (1868 CE). In lineage 3, the *Pro* region diverged around 50 years ago (1966 CE) and the *RdRp* region around 80 years ago (1936 CE). Ozaki et al. (2018) recently classified 23 ORF1 genotypes in the HuNoV GII strains into three lineages: lineage 1 (GII.P6-P8, P15 and P20), lineage 2 (GII.P1-P5, P12, P16, P17, P21, P30-P33, P35, and P37), and lineage 3 (GII.P22, P23, and P24). The phylogenetic topology in the ORF1 genotypes and the year of divergence of each lineage were almost analogous between the *Pro* and the *RdRp* regions. Thus, we estimated that the *Pro* and *RdRp* regions undergo shared evolution without recombination in NoV GII. However, we found incompatibility between the topology and the divergent year between the *Pro* and the *VP1* regions (Kobayashi et al., 2016), suggesting distinct evolutionary patterns with recombination between the *Pro/RdRp* region and the *VP1* region in NoV GII.

Several evolutionary studies of the *VP1* region and the *RdRp* region of NoVs have been carried out (Bok et al., 2009; Siebenga et al., 2010; Mahar et al., 2013; Kobayashi et al., 2016; Motoya et al., 2017). In a study of NoV protease, Cotten et al. (2014) estimated the evolutionary rate of the 3CL-Pro in GII.P4-GII.4 as 6.03 × 10^–3^ substitutions/site/year. However, there appears to be no comprehensive evolutionary studies using strains collected globally. This study showed that the evolutionary rate of the GII *Pro* region was estimated at 3.94 × 10^–3^ substitutions/site/year, and that evolution at different rates occurred between the ORF1 genotypes (Figure 2). The *Pro* region evolved rapidly (>10^–3^ substitutions/site/year) as did the *VP1* region and the *RdRp* region (Kobayashi et al., 2016; Ozaki et al., 2018). We therefore speculate that evolutionary rates in the other regions on the NoV genome were rapid.

We analyzed the phylogenetic distances among strains of each genotype, and discovered that the distances differed among strains for each genotype (Figures 4A–H). The histograms of the distances of the GII.P4 and GII.P7 indicated a broad range of distributions, suggesting that they are more genetically diverse than the other ORF1 genotypes. The histograms of the distances of GII.P2, GII.P12, GII.P17, GII.P21, and GII.P31 showed a narrow range distribution, suggesting low genetic divergence. The histogram of the distance of GII.P16 showed a bimodal distribution, suggesting that they contain two distinct groups within the same genotype. The trends of phylogenetic distance distribution among genotypes in the GII *Pro* region were analogous to that of the GII *RdRp* region (Ozaki et al., 2018). Thus, it appears that the GII *Pro* and *RdRp* regions might co-evolve.

We also predicted negative selection sites in the Pro protein *in silico*, and mapped these sites (Figure 5). We identified several possible negative selection sites in the Pro proteins of each genotype. Of them, the GII.P4 Pro protein contained more negative selection sites (20 sites per monomer) at the amino acid substitution sites than other genotypes. The substitution sites under negative selection were separated from the active site of NoV GII protease (Figure 5). In general, negative selection could be important in preventing the loss of viral protein function, because a high proportion of mutations will be deleterious (Domingo, 2006). These results may suggest that the amino acid substitutions distant from the active site in the GII Pro proteins are deleterious for viral propagation, probably, due to instability of the protein structure. We also found that relatively many amino acid substitutions occurred near the active site of the GII Pro proteins (Figure 5, Supplementary Table S3 and Supplementary Figure S2). These substitutions were mostly predicted to be under neutral selection. Thus, the substitutions may not influence the loss of the GII Pro protein function but may change the cleavage activity and affect the efficiency of viral propagation. A previous study reported that the amino acids adjacent to the active sites in the Pro protein of enterovirus 71 play a crucial role in protease activity based on the reduction of activity caused by the mutations of the residues (Wang et al., 2011). Moreover, Viskovska et al. (2019) reported that the NoV GII protease showed activity in a pH-dependent manner by interactions between the residues H30 and R112. In this study, we found that these amino acids were conserved in most GII genotypes, except for GII.P25 (Supplementary Table S3). Therefore, these results suggest that the GII genotypes have a potential to exhibit pH-dependent protease activity.

The phylodynamics of each ORF1 genotype were also assessed in this study (Figure 6). The phylodyamics of GII.P4, GII.P7, GII.P16, GII.P21, and GII.P31 fluctuated at specific times. GII.P4 increased around 2005 and 2008. GII.P7, GII.P16, GII.P21, and GII.P31 increased around 2012, 2015–2016, 2014–2015, and 2011–2012, respectively. In GII.P4 and GII.P31, the collection year of GII.4 agreed with the year when the population size increased. In GII.P7 and GII.P21, the collection year of GII.6 and GII.3, respectively, was almost consistent with the year in which the population size increased. In GII.P16, the collection year of GII.2 and GII.4 corresponded with the year of increase of population size (Nagasawa et al., 2018; Barclay et al., 2019). Previous reports have shown a correlation between fluctuations in population size and the epidemiology of actual outbreaks (Motomura et al., 2008, 2010; Chen et al., 2015; van Beek et al., 2017; Nagasawa et al., 2018; Barclay et al., 2019; Brown et al., 2019). In this study, we observed similar results in the phylodynamics of NoV protease according to the *VP1* and *RdRp* regions (Kobayashi et al., 2016; Ozaki et al., 2018). These results suggest that it is useful to analyze phylodynamics focused on the *Pro* region in order to investigate past epidemics of each NoV genotype. This study has a limitation, in that it used a restricted number of GII *Pro* sequences from the GenBank. In addition, sequences with ≥99.8% identity were omitted. These selection biases may have affected the results of the bioinformatics analyses to some extent.

## Conclusion

In conclusion, we estimated that the common ancestor of the GII *Pro* region diverged many years ago, and NoV GII evolved rapidly. The GII *Pro* regions and proteins evolved with many amino acid substitutions, but no positively selective pressure was identified. The substitutions under neutral selection adjacent to the active sites of the proteins suggest that the GII genotypes have a potential to alter the protease activity. This study indicates a need to compare the efficiency of antiviral drugs for the Pro protein between NoV genotypes in conjunction with continuing investigation into the evolution of the NoV *Pro* region and the activity of the protein *in vitro*.

## Data Availability Statement

All datasets generated for this study are included in the article/Supplementary Material.

## Author Contributions

KO, KK, and HK designed the study. KO, YM, KN, JA, TS, KY, KM, TM, MK, and HK analyzed the data. KO, YM, AR, KK, and HK wrote and supervised the study. All authors read and approved the manuscript.

## Conflict of Interest

KO was employed by the company Niitaka Co., Ltd. The remaining authors declare that the research was conducted in the absence of any commercial or financial relationships that could be construed as a potential conflict of interest.

## Footnotes

1 http://www.ncbi.nlm.nih.gov/genbank

2 http://tree.bio.ed.ac.uk/software/tracer/

3 http://tree.bio.ed.ac.uk/software/figtree/
