# Supplementary material for: Molecular Evolution of the Protease Region in Norovirus Genogroup II
Source: Front Microbiol. 2020 Jan 14;10:2991. doi: 10.3389/fmicb.2019.02991 (PMC6971112; doi:10.3389/fmicb.2019.02991)
Supplement: Supplementary file 1 [file Data_Sheet_1.PDF]

Supplementary Material

Molecular Evolution of the Protease Region in Norovirus Genogroup II

Keita Ozaki, Yuki Matsushima, Koo Nagasawa, Jumpei Aso, Takeshi Saraya, Keisuke Yoshihara, Koichi Murakami, Takumi Motoya, Akihide Ryo, Makoto Kuroda, Kazuhiko Katayama* and Hirokazu Kimura*

*** Correspondence:**

Hirokazu Kimura: h-kimura@paz.ac.jp

Kazuhiko Katayama: katayama@lisci.kitasato-u.ac.jp

Supplementary Figures

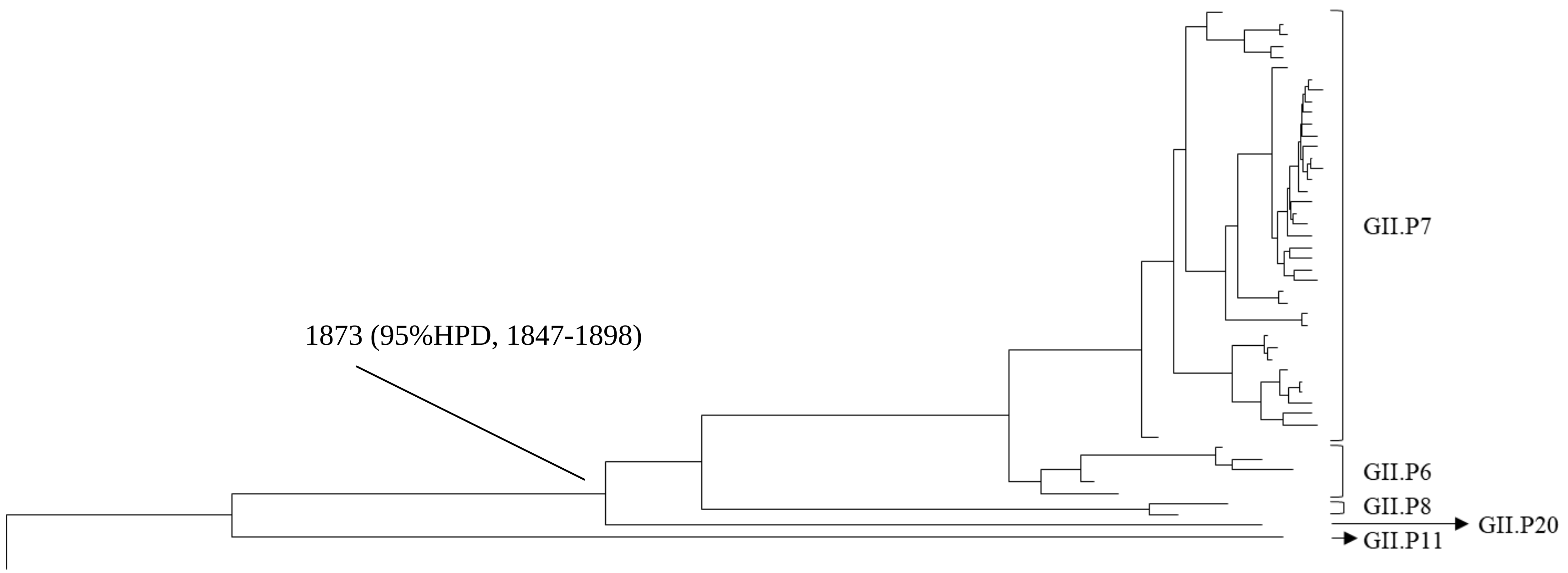

Figure S1. Time scaled phylogenetic tree of the NoV *Pro* region constructed by the Bayesian MCMC method. Enlarged tree focused on the lineage 1 (GII.P6, P7, P8 and P20).

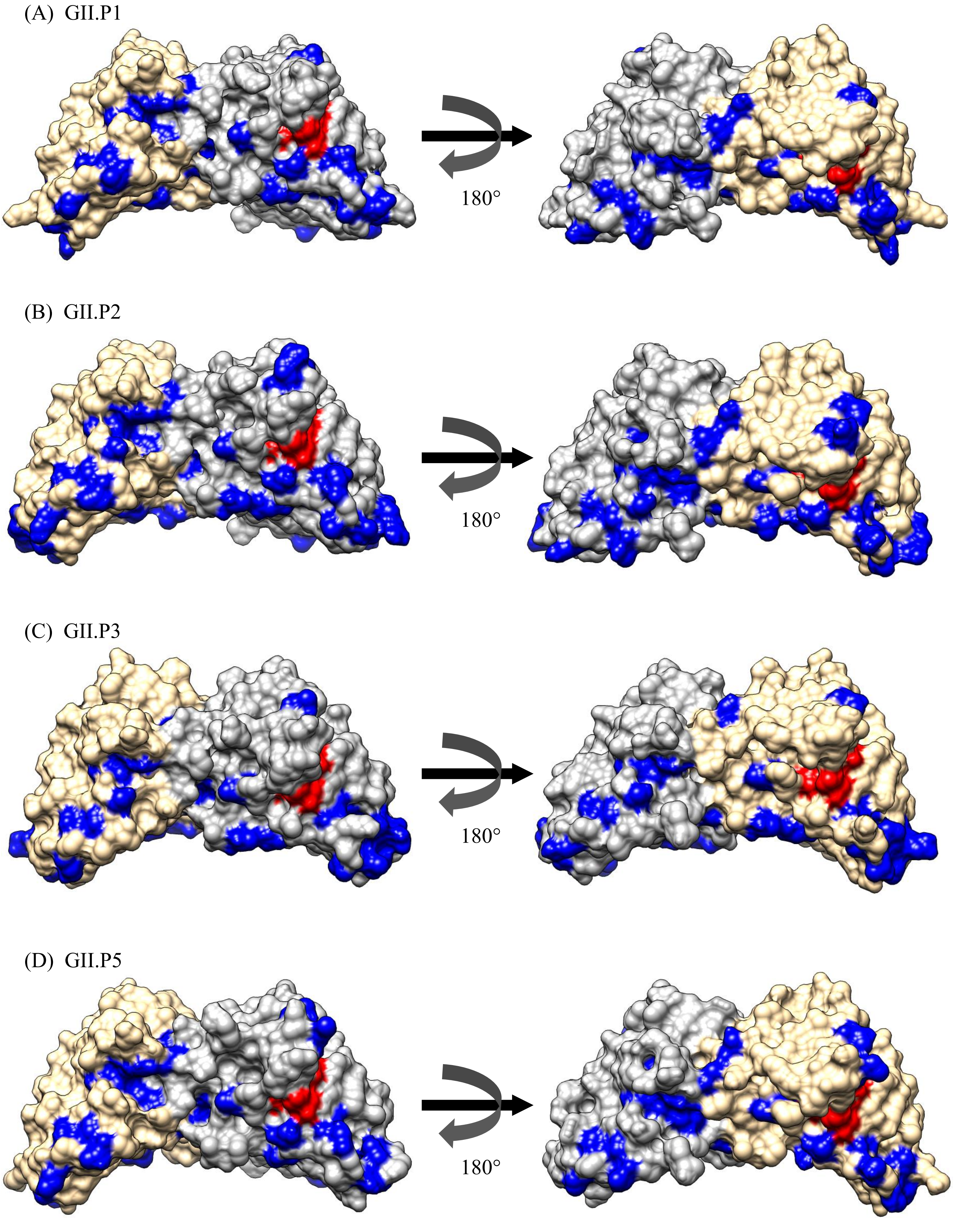
(A) GII.P1
180°
(B) GII.P2
180°
(C) GII.P3
180°
(D) GII.P5
180°

(E) GII.P6

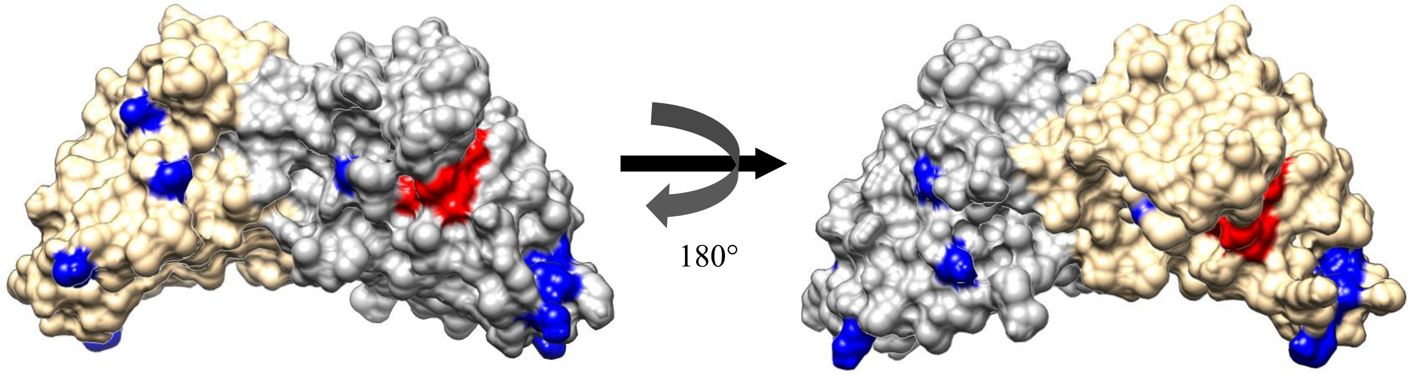

(F) GII.P8

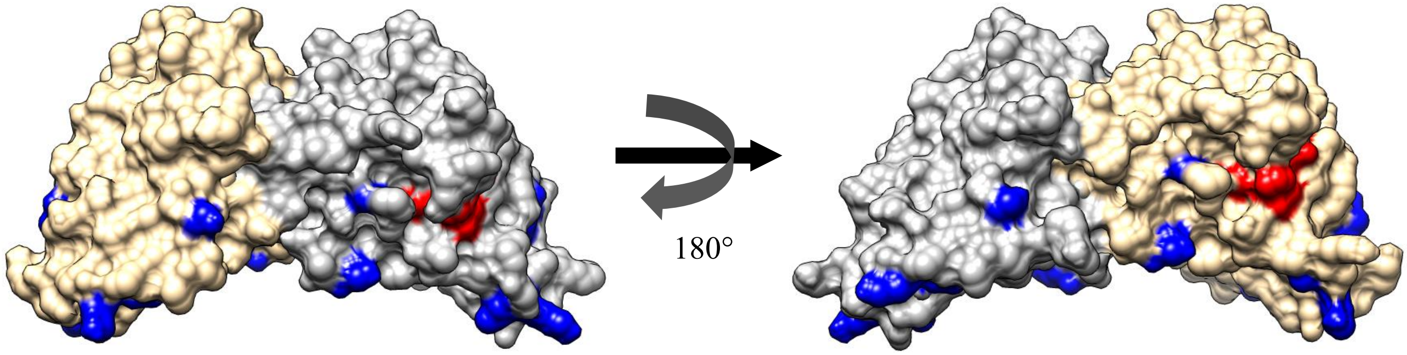

(G) GII.P17

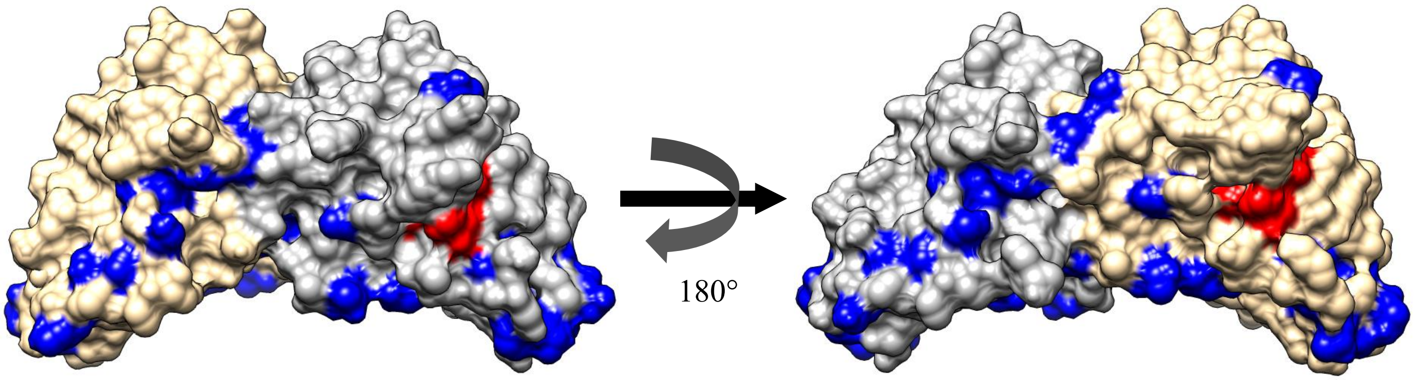

(H) GII.P24

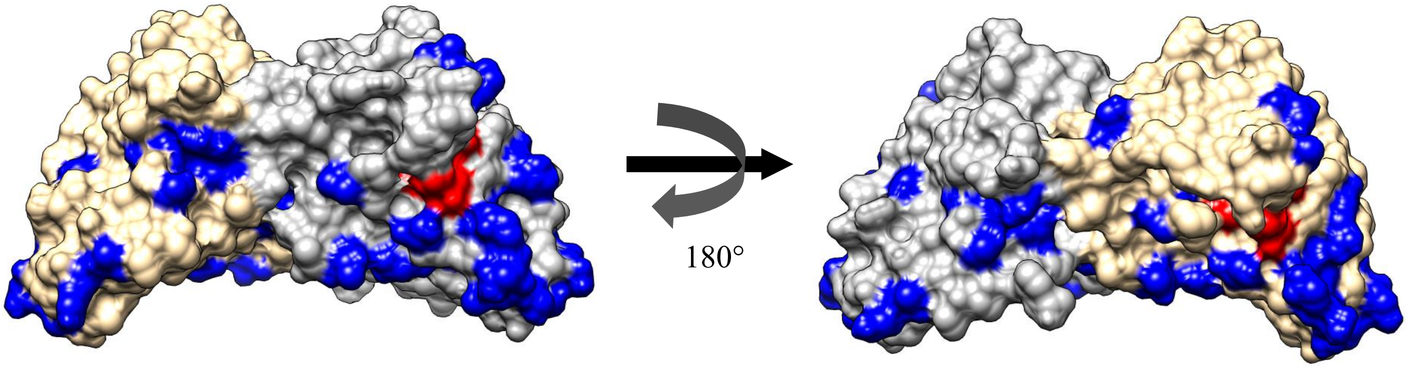

(I) GII.P25

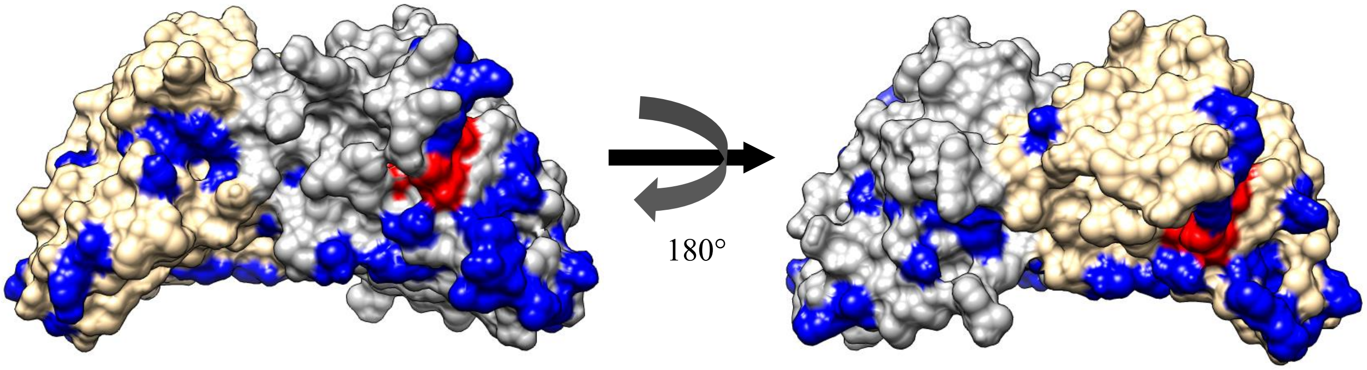

(J) GII.P30

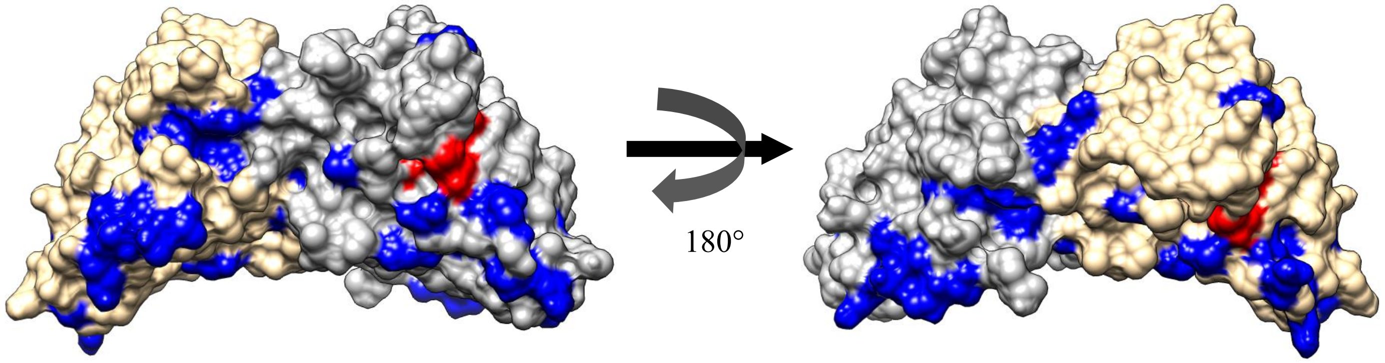

(K) GII.P32

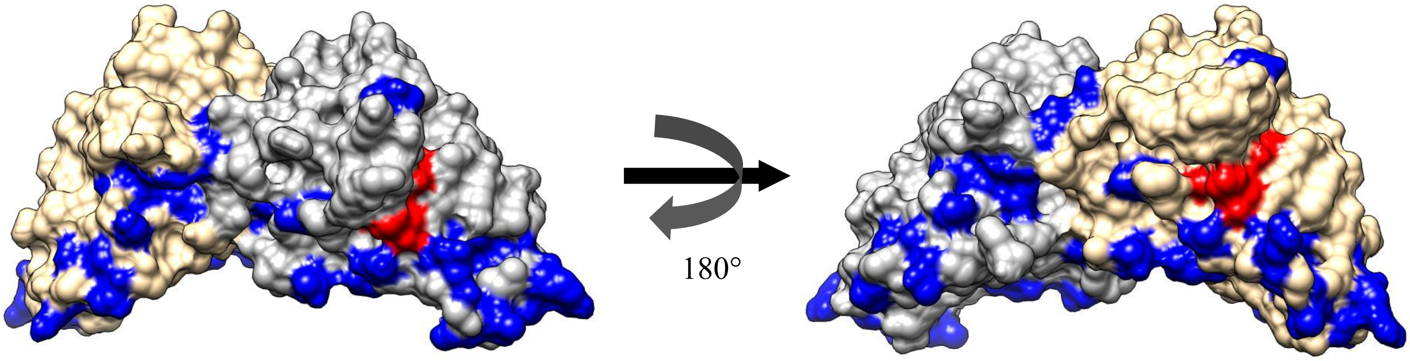

(L) GII.P33

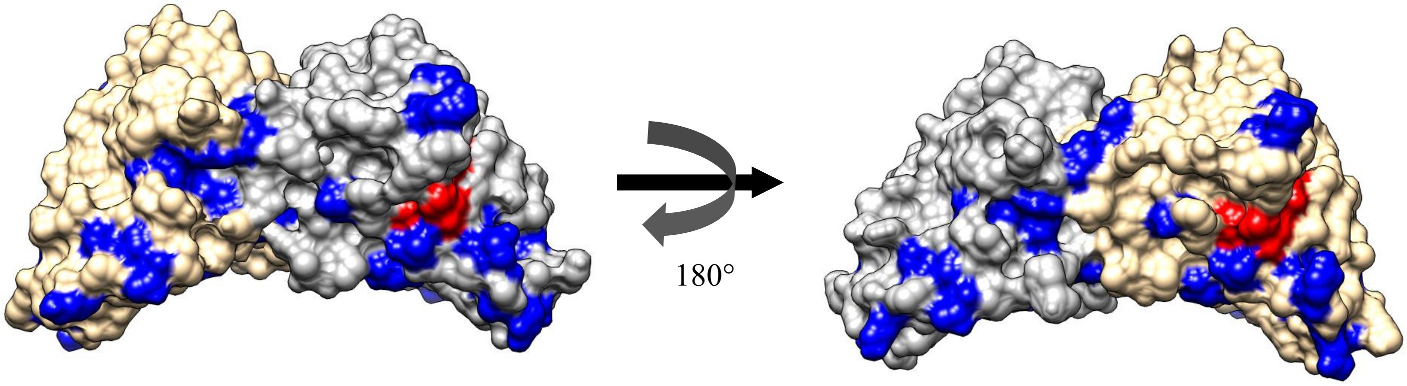

(M) GII.P35

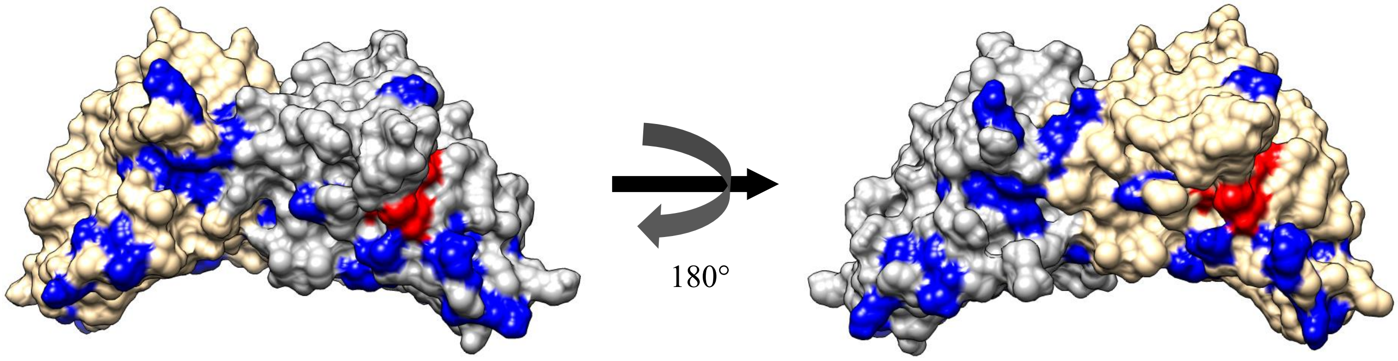

(N) GII.P37

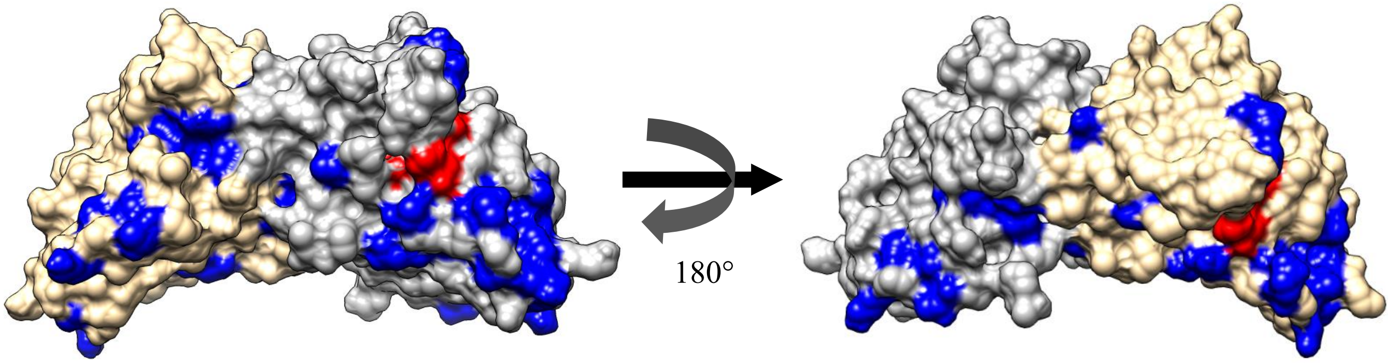

(O) GII.P39

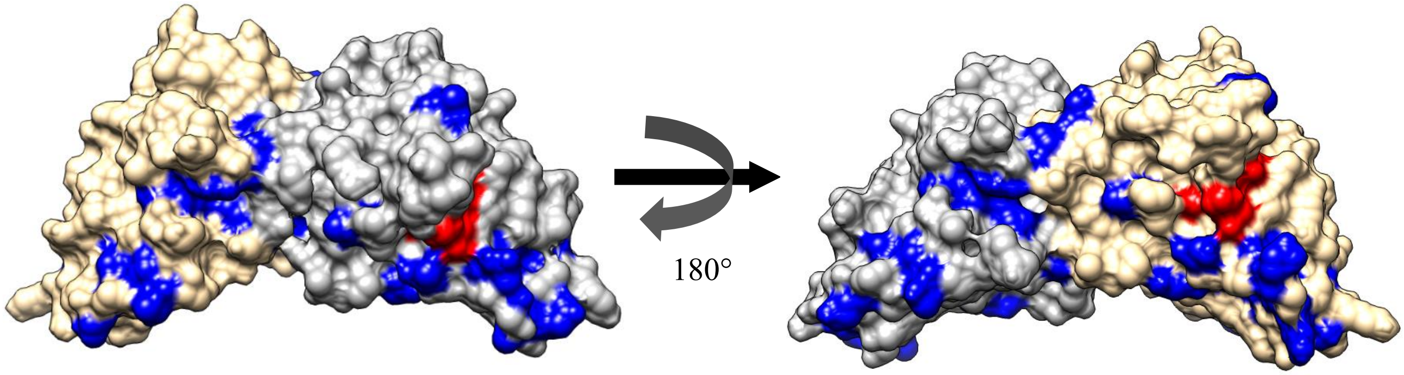

(P) GII.P40

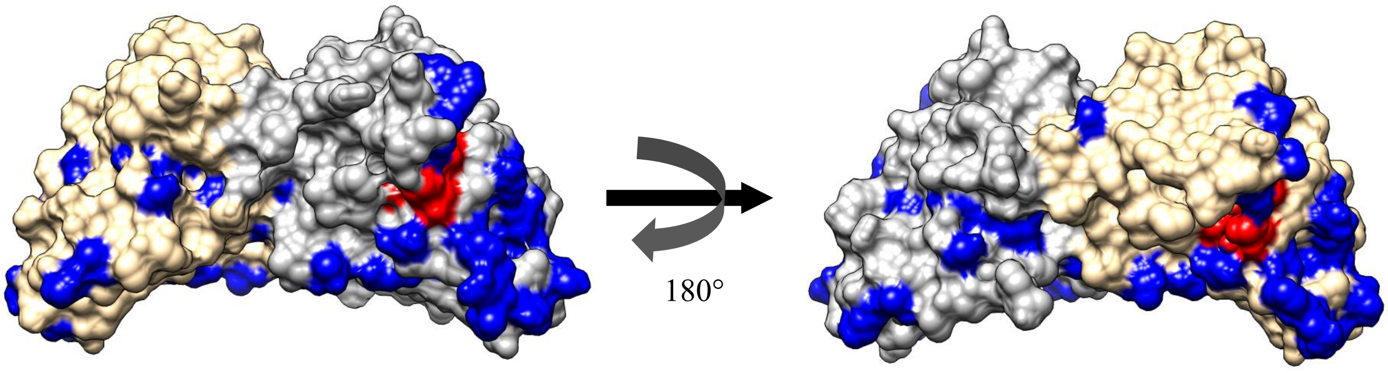

(Q) GII.P41

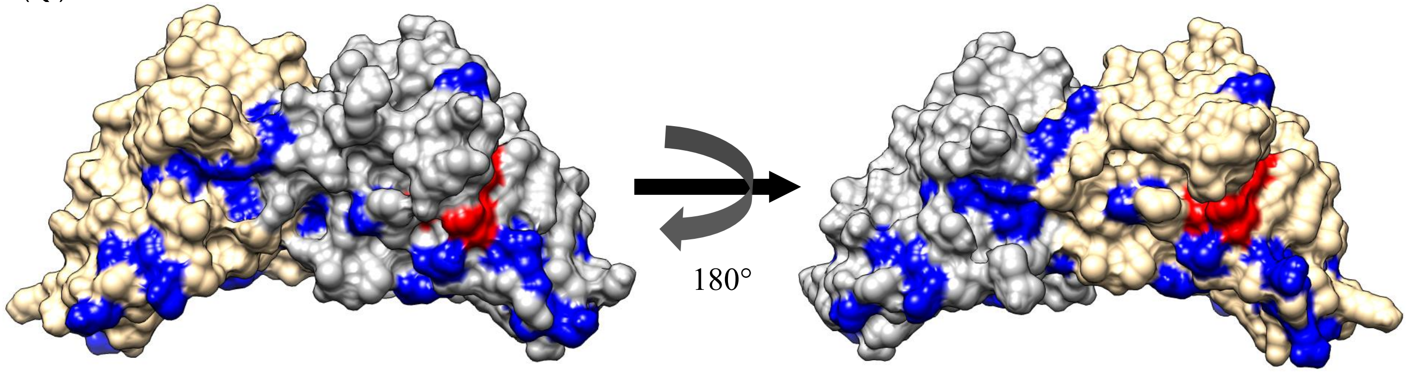

(R) GII.PNA5

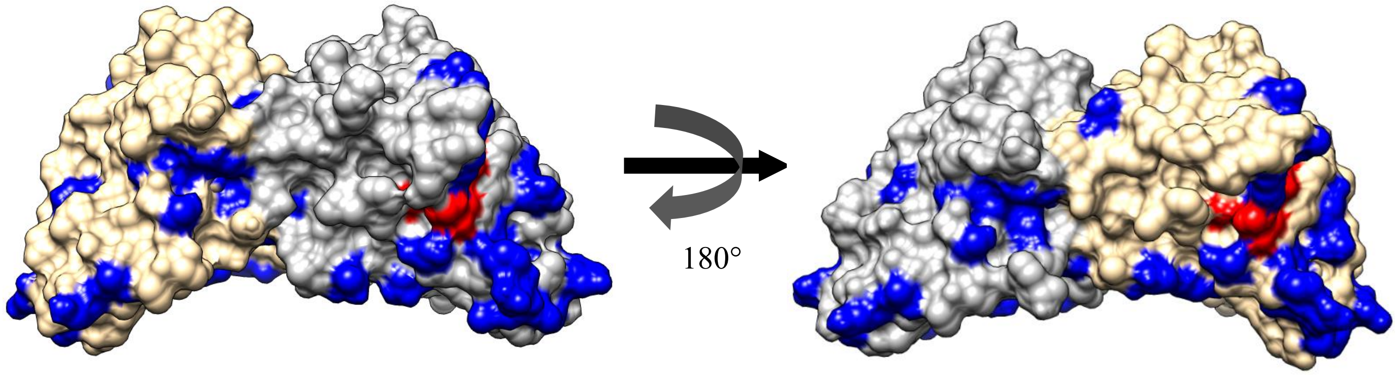

(S) GII.PNA7

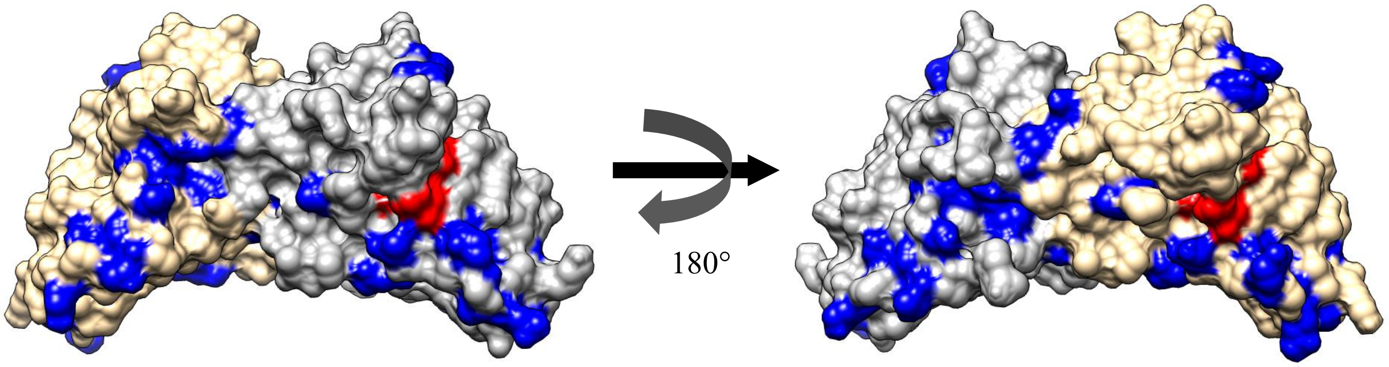

Figure S2. Structural models for the Pro protein of each genotype. Three-dimensional Pro dimer structures for GII.P1 (A), GII.P2 (B), GII.P3 (C), GII.P5 (D), GII.6 (E), GII.P8 (F), GII.P17 (G), GII.P24 (H), GII.P25 (I), GII.P30 (J), GII.P32 (K), GII.P33 (L), GII.P35 (M), GII.P37 (N), GII.P39 (O), GII.P40 (P), GII.P41 (Q), GII.PNA5 (R) and GII.PNA7 (S) are shown. The chains to be composed of the dimer structures are coloured grey (chain A) and Navajo white (chain B). Amino acid substitutions of the other genotypes compared to a GII.P20 strain are coloured blue. Active site residues are coloured red.

Supplemental Tables

Table S1. The strains used in this study

GenBank accession No.	ORF1 genotype	Name
U07611	GII.P1	GII/Hu/US/1971/GII.1[P1]/Hawaii
LC209438	GII.P2	GII/Hu/JP/2004/GII.2[P2]/Tochigi-86
LC209440	GII.P2	GII/Hu/JP/2015/GII.2[P2]/Saitama-169
LC209457	GII.P2	GII/Hu/JP/2015/GII.2[P2]/Miyagi-63
LC209462	GII.P2	GII/Hu/JP/2006/GII.2[P2]/Hokkaido-14
LC209463	GII.P2	GII/Hu/JP/2008/GII.2[P2]/Hokkaido-15
LC209465	GII.P2	GII/Hu/JP/2010/GII.2[P2]/Hokkaido-16
LC209469	GII.P2	GII/Hu/JP/2014/GII.2[P2]/Yamaguchi-014
MH218655	GII.P2	GII/Hu/UK/2015/GII.2[P2]/NORO_187_01_10
MH218734	GII.P2	GII/Hu/UK/2015/GII.2[P2]/NORO_95_25_01
MH218735	GII.P2	GII/ Hu/UK/2015/GII.2[P2]/NORO_96_09_03
AB039782	GII.P3	GII/Hu/JP/1998/GII.3[P3]/SaitamaU201
KJ194500	GII.P3	GII/Hu/NL/1995/GII.3[P3]/Amsterdam/1
KJ194504	GII.P3	GII/Hu/NL/1995/GII.3[P3]/Amsterdam
AB541268	GII.P4 (Apeldoorn 2007)	GII/Hu/JP/2009/GII.4[P4]/Hokkaido5
AB541272	GII.P4 (Apeldoorn 2007)	GII/Hu/JP/2009/GII.4[P4]/Iwate3
AB541275	GII.P4 (Apeldoorn 2007)	GII/Hu/JP/2008/GII.4[P4]/Iwate5
AB541312	GII.P4 (Apeldoorn 2007)	GII/Hu/JP/2008/GII.4[P4]/Niigata2
AB541318	GII.P4 (Apeldoorn 2007)	GII/Hu/JP/2009/GII.4[P4]/Niigata5
AB541320	GII.P4 (Apeldoorn 2007)	GII/Hu/JP/2008/GII.4[P4]/Osaka1
AB933729	GII.P4 (Apeldoorn 2007)	GII/Hu/JP/2009/GII.4[P4]/Fukui5
AB933730	GII.P4 (Apeldoorn 2007)	GII/Hu/JP/2009/GII.4[P4]/Ehime1
AB933759	GII.P4 (Apeldoorn 2007)	GII/Hu/JP/20011/GII.4[P4]/Nagano26
HM748973	GII.P4 (Apeldoorn 2007)	GII/Hu/AU/2009/GII.4[P4]/NSW892U
HQ009513	GII.P4 (Apeldoorn 2007)	GII/Hu/KR/2008/GII.4[P4]/JB-15
JX439815	GII.P4 (Apeldoorn 2007)	GII/Hu/KR/2010/GII.4[P4]/Seoul1055
JX439816	GII.P4 (Apeldoorn 2007)	GII/Hu/KR/2010/GII.4[P4]/Seoul1072
JX439818	GII.P4 (Apeldoorn 2007)	GII/Hu/KR/2010/GII.4[P4]/Seoul1367
JX445161	GII.P4 (Apeldoorn 2007)	GII/Hu/CA/2008/GII.4[P4]/AlbertaEl210
JX459903	GII.P4 (Apeldoorn 2007)	GII/Hu/AU/2011/GII.4[P4]/Jannali/NSW774M
KC409311	GII.P4 (Apeldoorn 2007)	GII/Hu/VN/2009/GII.4[P4]/30199
KF429791	GII.P4 (Apeldoorn 2007)	GII/Hu/US/2011/GII.4[P4]/NIHIC1.5
KF712507	GII.P4 (Apeldoorn 2007)	GII/Hu/US/2012/GII.4[P4]/NIHIC1.11
MF140633	GII.P4 (Apeldoorn 2007)	GII/Hu/NL/2009/GII.4[P4]/Rotterdam/E7800007
MF140634	GII.P4 (Apeldoorn 2007)	GII/Hu/NL/2010/GII.4[P4]/Rotterdam/E1300272
MF140635	GII.P4 (Apeldoorn 2007)	GII/Hu/NL/2010/GII.4[P4]/Rotterdam/E7800016
FJ537137	GII.P4 (Bristol 1993)	GII/Hu/US/1987/GII.4[P4]/CHDC4108
X86557	GII.P4 (Bristol 1993)	GII/Hu/UK/1993/GII.4[P4]/Lordsdale
AB985442	GII.P4 (Camberwell 1994)	GII/Hu/JP/1987/GII.4[P4]/2-46/Tokyo
AY032605	GII.P4 (Camberwell 1994)	GII/Hu/US/1987/GII.4[P4]/MD145-12
JX289821	GII.P4 (Camberwell 1994)	GII/Hu/US/1987/GII.4[P4]/MD120-12
KY424332	GII.P4 (Camberwell 1994)	GII/Hu/US/1987/GII.4[P4]/MD104-2
KY424334	GII.P4 (Camberwell 1994)	GII/Hu/US/1987/GII.4[P4]/MD104-7
KY424337	GII.P4 (Camberwell 1994)	GII/Hu/US/1987/GII.4[P4]/MD143-4
KY424339	GII.P4 (Camberwell 1994)	GII/Hu/US/1988/GII.4[P4]/MD04-1A
AB447427	GII.P4 (Den Haag 2006b)	GII/Hu/JP/2006/GII.4[P4]/Hokkaido1
AB447428	GII.P4 (Den Haag 2006b)	GII/Hu/JP/2006/GII.4[P4]/Hokkaido2
AB447429	GII.P4 (Den Haag 2006b)	GII/Hu/JP/2006/GII.4[P4]/Hokkaido3
AB447430	GII.P4 (Den Haag 2006b)	GII/Hu/JP/2006/GII.4[P4]/Hokkaido4
AB447431	GII.P4 (Den Haag 2006b)	GII/Hu/JP/2007/GII.4[P4]/Hokkaido5
AB447436	GII.P4 (Den Haag 2006b)	GII/Hu/JP/2006/GII.4[P4]/Akita1
AB447438	GII.P4 (Den Haag 2006b)	GII/Hu/JP/2006/GII.4[P4]/Akita4
AB447439	GII.P4 (Den Haag 2006b)	GII/Hu/JP/2006/GII.4[P4]/Akita5
AB447443	GII.P4 (Den Haag 2006b)	GII/Hu/JP/2006/GII.4[P4]/Toyama1
AB447445	GII.P4 (Den Haag 2006b)	GII/Hu/JP/2006/GII.4[P4]/Toyama5
AB447449	GII.P4 (Den Haag 2006b)	GII/Hu/JP/2006/GII.4[P4]/Sakai3
AB447451	GII.P4 (Den Haag 2006b)	GII/Hu/JP/2006/GII.4[P4]/Hiroshima1

Table S1 (continued). The strains used in this study.

GenBank accession No.	ORF1 genotype	Name
AB447453	GII.P4 (Den Haag 2006b)	GII/Hu/JP/2006/GII.4[P4]/Ehime1
AB447455	GII.P4 (Den Haag 2006b)	GII/Hu/JP/2006/GII.4[P4]/Ehime5
AB447456	GII.P4 (Den Haag 2006b)	GII/Hu/JP/2006/GII.4[P4]/Saga1
AB541201	GII.P4 (Den Haag 2006b)	GII/Hu/JP/2008/GII.4[P4]/Aichi1
AB541212	GII.P4 (Den Haag 2006b)	GII/Hu/JP/2008/GII.4[P4]/Akita1
AB541213	GII.P4 (Den Haag 2006b)	GII/Hu/JP/2008/GII.4[P4]/Akita2
AB541214	GII.P4 (Den Haag 2006b)	GII/Hu/JP/2007/GII.4[P4]/Akita3
AB541217	GII.P4 (Den Haag 2006b)	GII/Hu/JP/2007/GII.4[P4]/Akita5
AB541220	GII.P4 (Den Haag 2006b)	GII/Hu/JP/2008/GII.4[P4]/Aomori2
AB541222	GII.P4 (Den Haag 2006b)	GII/Hu/JP/2008/GII.4[P4]/Aomori3
AB541228	GII.P4 (Den Haag 2006b)	GII/Hu/JP/2007/GII.4[P4]/Chiba1
AB541230	GII.P4 (Den Haag 2006b)	GII/Hu/JP/2007/GII.4[P4]/Chiba2
AB541232	GII.P4 (Den Haag 2006b)	GII/Hu/JP/2007/GII.4[P4]/Chiba4
AB541234	GII.P4 (Den Haag 2006b)	GII/Hu/JP/2008/GII.4[P4]/Chiba5
AB541237	GII.P4 (Den Haag 2006b)	GII/Hu/JP/2008/GII.4[P4]/Ehime1
AB541240	GII.P4 (Den Haag 2006b)	GII/Hu/JP/2009/GII.4[P4]/Ehime3
AB541241	GII.P4 (Den Haag 2006b)	GII/Hu/JP/2008/GII.4[P4]/Ehime4
AB541242	GII.P4 (Den Haag 2006b)	GII/Hu/JP/2009/GII.4[P4]/Ehime4
AB541244	GII.P4 (Den Haag 2006b)	GII/Hu/JP/2008/GII.4[P4]/Fukui1
AB541245	GII.P4 (Den Haag 2006b)	GII/Hu/JP/2007/GII.4[P4]/Fukui2
AB541247	GII.P4 (Den Haag 2006b)	GII/Hu/JP/2007/GII.4[P4]/Fukui4
AB541248	GII.P4 (Den Haag 2006b)	GII/Hu/JP/2008/GII.4[P4]/Fukui4
AB541249	GII.P4 (Den Haag 2006b)	GII/Hu/JP/2007/GII.4[P4]/Fukui5
AB541250	GII.P4 (Den Haag 2006b)	GII/Hu/JP/2008/GII.4[P4]/Fukui5
AB541252	GII.P4 (Den Haag 2006b)	GII/Hu/JP/2008/GII.4[P4]/Hiroshima1
AB541254	GII.P4 (Den Haag 2006b)	GII/Hu/JP/2008/GII.4[P4]/Hiroshima2
AB541256	GII.P4 (Den Haag 2006b)	GII/Hu/JP/2008/GII.4[P4]/Hiroshima3
AB541259	GII.P4 (Den Haag 2006b)	GII/Hu/JP/2009/GII.4[P4]/Hiroshima5
AB541262	GII.P4 (Den Haag 2006b)	GII/Hu/JP/2007/GII.4[P4]/Hokkaido2
AB541263	GII.P4 (Den Haag 2006b)	GII/Hu/JP/2008/GII.4[P4]/Hokkaido2
AB541265	GII.P4 (Den Haag 2006b)	GII/Hu/JP/2008/GII.4[P4]/Hokkaido4
AB541276	GII.P4 (Den Haag 2006b)	GII/Hu/JP/2008/GII.4[P4]/Iwate5
AB541279	GII.P4 (Den Haag 2006b)	GII/Hu/JP/2007/GII.4[P4]/Kumamoto3
AB541280	GII.P4 (Den Haag 2006b)	GII/Hu/JP/2007/GII.4[P4]/Kumamoto4
AB541282	GII.P4 (Den Haag 2006b)	GII/Hu/JP/2007/GII.4[P4]/Miyagi2
AB541290	GII.P4 (Den Haag 2006b)	GII/Hu/JP/2007/GII.4[P4]/Miyazaki2
AB541292	GII.P4 (Den Haag 2006b)	GII/Hu/JP/2008/GII.4[P4]/Miyazaki3
AB541297	GII.P4 (Den Haag 2006b)	GII/Hu/JP/2008/GII.4[P4]/Miyazaki6
AB541303	GII.P4 (Den Haag 2006b)	GII/Hu/JP/2007/GII.4[P4]/Nagano2
AB541305	GII.P4 (Den Haag 2006b)	GII/Hu/JP/2007/GII.4[P4]/Nagano3
AB541306	GII.P4 (Den Haag 2006b)	GII/Hu/JP/2009/GII.4[P4]/Nagano3
AB541307	GII.P4 (Den Haag 2006b)	GII/Hu/JP/2009/GII.4[P4]/Nagano4
AB541308	GII.P4 (Den Haag 2006b)	GII/Hu/JP/2007/GII.4[P4]/Nagano5
AB541309	GII.P4 (Den Haag 2006b)	GII/Hu/JP/2007/GII.4[P4]/Niigata1
AB541311	GII.P4 (Den Haag 2006b)	GII/Hu/JP/2007/GII.4[P4]/Niigata2
AB541314	GII.P4 (Den Haag 2006b)	GII/Hu/JP/2009/GII.4[P4]/Niigata3
AB541316	GII.P4 (Den Haag 2006b)	GII/Hu/JP/2009/GII.4[P4]/Niigata4
AB541324	GII.P4 (Den Haag 2006b)	GII/Hu/JP/2008/GII.4[P4]/Osaka3
AB541326	GII.P4 (Den Haag 2006b)	GII/Hu/JP/2008/GII.4[P4]/Osaka4
AB541329	GII.P4 (Den Haag 2006b)	GII/Hu/JP/2008/GII.4[P4]/Osaka6
AB541332	GII.P4 (Den Haag 2006b)	GII/Hu/JP/2006/GII.4[P4]/Saga2
AB541334	GII.P4 (Den Haag 2006b)	GII/Hu/JP/2009/GII.4[P4]/Saga3
AB541337	GII.P4 (Den Haag 2006b)	GII/Hu/JP/2007/GII.4[P4]/Saga5
AB541340	GII.P4 (Den Haag 2006b)	GII/Hu/JP/2008/GII.4[P4]/Sakai1
AB541343	GII.P4 (Den Haag 2006b)	GII/Hu/JP/2008/GII.4[P4]/Sakai3
AB541346	GII.P4 (Den Haag 2006b)	GII/Hu/JP/2007/GII.4[P4]/Shimane1
AB541352	GII.P4 (Den Haag 2006b)	GII/Hu/JP/2007/GII.4[P4]/Shimane5
AB541353	GII.P4 (Den Haag 2006b)	GII/Hu/JP/2009/GII.4[P4]/Shimane5
AB541354	GII.P4 (Den Haag 2006b)	GII/Hu/JP/2007/GII.4[P4]/Toyama1
AB541355	GII.P4 (Den Haag 2006b)	GII/Hu/JP/2007/GII.4[P4]/Toyama2

Table S1 (continued). The strains used in this study.

GenBank accession No.	ORF1 genotype	Name
AB541360	GII.P4 (Den Haag 2006b)	GII/Hu/JP/2008/GII.4[P4]/Toyama4
AB541361	GII.P4 (Den Haag 2006b)	GII/Hu/JP/2008/GII.4[P4]/Toyama5
AB933649	GII.P4 (Den Haag 2006b)	GII/Hu/JP/2009/GII.4[P4]/Hiroshimacity3
AB933651	GII.P4 (Den Haag 2006b)	GII/Hu/JP/2009/GII.4[P4]/Hiroshimacity5
AB933653	GII.P4 (Den Haag 2006b)	GII/Hu/JP/2009/GII.4[P4]/Aichi1
AB933655	GII.P4 (Den Haag 2006b)	GII/Hu/JP/2009/GII.4[P4]/Shimane2
AB933656	GII.P4 (Den Haag 2006b)	GII/Hu/JP/2009/GII.4[P4]/Miyazaki1
AB933660	GII.P4 (Den Haag 2006b)	GII/Hu/JP/2010/GII.4[P4]/Toyama5
AB933661	GII.P4 (Den Haag 2006b)	GII/Hu/JP/2010/GII.4[P4]/Chiba1
AB933663	GII.P4 (Den Haag 2006b)	GII/Hu/JP/2010/GII.4[P4]/Chiba5
AB933664	GII.P4 (Den Haag 2006b)	GII/Hu/JP/2010/GII.4[P4]/Nagano1
AB933666	GII.P4 (Den Haag 2006b)	GII/Hu/JP/2010/GII.4[P4]/Osaka2
AB933667	GII.P4 (Den Haag 2006b)	GII/Hu/JP/2010/GII.4[P4]/Osaka3
AB933668	GII.P4 (Den Haag 2006b)	GII/Hu/JP/2010/GII.4[P4]/Osaka4
AB933670	GII.P4 (Den Haag 2006b)	GII/Hu/JP/2010/GII.4[P4]/Sakai2
AB933671	GII.P4 (Den Haag 2006b)	GII/Hu/JP/2010/GII.4[P4]/Hiroshima3
AB933672	GII.P4 (Den Haag 2006b)	GII/Hu/JP/2010/GII.4[P4]/Hiroshima4
AB933675	GII.P4 (Den Haag 2006b)	GII/Hu/JP/2010/GII.4[P4]/Hokkaido4
AB933677	GII.P4 (Den Haag 2006b)	GII/Hu/JP/2010/GII.4[P4]/Niigata3
AB933678	GII.P4 (Den Haag 2006b)	GII/Hu/JP/2010/GII.4[P4]/Niigata4
AB933679	GII.P4 (Den Haag 2006b)	GII/Hu/JP/2010/GII.4[P4]/Fukui2
AB933680	GII.P4 (Den Haag 2006b)	GII/Hu/JP/2010/GII.4[P4]/Fukui3
AB933682	GII.P4 (Den Haag 2006b)	GII/Hu/JP/2010/GII.4[P4]/Nagano3
AB933684	GII.P4 (Den Haag 2006b)	GII/Hu/JP/2010/GII.4[P4]/Sakai4
AB933685	GII.P4 (Den Haag 2006b)	GII/Hu/JP/2010/GII.4[P4]/Ehime3
AB933690	GII.P4 (Den Haag 2006b)	GII/Hu/JP/2010/GII.4[P4]/Miyazaki4
AB933693	GII.P4 (Den Haag 2006b)	GII/Hu/JP/2011/GII.4[P4]/Sakai7
AB933697	GII.P4 (Den Haag 2006b)	GII/Hu/JP/2011/GII.4[P4]/Shimane2
AB933698	GII.P4 (Den Haag 2006b)	GII/Hu/JP/2011/GII.4[P4]/Hiroshimacity2
AB933699	GII.P4 (Den Haag 2006b)	GII/Hu/JP/2011/GII.4[P4]/Akita2
AB933703	GII.P4 (Den Haag 2006b)	GII/Hu/JP/2011/GII.4[P4]/Shimane4
AB933704	GII.P4 (Den Haag 2006b)	GII/Hu/JP/2011/GII.4[P4]/Ehime1
AB933706	GII.P4 (Den Haag 2006b)	GII/Hu/JP/2011/GII.4[P4]/Saga1
AB933709	GII.P4 (Den Haag 2006b)	GII/Hu/JP/2011/GII.4[P4]/Hokkaido1
AB933713	GII.P4 (Den Haag 2006b)	GII/Hu/JP/2011/GII.4[P4]/Aomori7
AB933714	GII.P4 (Den Haag 2006b)	GII/Hu/JP/2011/GII.4[P4]/Iwate2
AB933715	GII.P4 (Den Haag 2006b)	GII/Hu/JP/2011/GII.4[P4]/Iwate4
AB933716	GII.P4 (Den Haag 2006b)	GII/Hu/JP/2011/GII.4[P4]/Iwate5
AB933717	GII.P4 (Den Haag 2006b)	GII/Hu/JP/2011/GII.4[P4]/Toyama5
AB933718	GII.P4 (Den Haag 2006b)	GII/Hu/JP/2011/GII.4[P4]/Aichi1
AB933721	GII.P4 (Den Haag 2006b)	GII/Hu/JP/2011/GII.4[P4]/Hiroshima4
AB933724	GII.P4 (Den Haag 2006b)	GII/Hu/JP/2011/GII.4[P4]/Aichi3
EF684915	GII.P4 (Den Haag 2006b)	GII/Hu/AU/2006/GII.4[P4]/Shellharbour/NSW696T
FJ514242	GII.P4 (Den Haag 2006b)	GII/Hu/KR/2008/GII.4[P4]/CUK-3
GQ845024	GII.P4 (Den Haag 2006b)	GII/Hu/AU/2007/GII.4[P4]/Rathmines/NSW287R
GQ845366	GII.P4 (Den Haag 2006b)	GII/Hu/AU/2008/GII.4[P4]/Westmead/NSW3639
GU325839	GII.P4 (Den Haag 2006b)	GII/Hu/US/2009/GII.4[P4]/HS194
GU991353	GII.P4 (Den Haag 2006b)	GII/Hu/CN/2008/GII.4[P4]/Shanghai/SH2
JN400601	GII.P4 (Den Haag 2006b)	GII/Hu/TW/2006/GII.4[P4]/CGMH03
JN400602	GII.P4 (Den Haag 2006b)	GII/Hu/TW/2006/GII.4[P4]/CGMH04
JN400603	GII.P4 (Den Haag 2006b)	GII/Hu/TW/2006/GII.4[P4]/CGMH05
JN400606	GII.P4 (Den Haag 2006b)	GII/Hu/TW/2006/GII.4[P4]/CGMH08
JN400607	GII.P4 (Den Haag 2006b)	GII/Hu/TW/2006/GII.4[P4]/CGMH09
JN400608	GII.P4 (Den Haag 2006b)	GII/Hu/TW/2006/GII.4[P4]/CGMH10
JN400611	GII.P4 (Den Haag 2006b)	GII/Hu/TW/2007/GII.4[P4]/CGMH13
JN400614	GII.P4 (Den Haag 2006b)	GII/Hu/TW/2007/GII.4[P4]/CGMH16
JN400615	GII.P4 (Den Haag 2006b)	GII/Hu/TW/2007/GII.4[P4]/CGMH17
JN400616	GII.P4 (Den Haag 2006b)	GII/Hu/TW/2008/GII.4[P4]/CGMH18
JN400617	GII.P4 (Den Haag 2006b)	GII/Hu/TW/2009/GII.4[P4]/CGMH19
JQ613572	GII.P4 (Den Haag 2006b)	GII/Hu/AU/2010/GII.4[P4]/StVincents/NSW217

Table S1 (continued). The strains used in this study.

GenBank accession No.	ORF1 genotype	Name
JQ622197	GII.P4 (Den Haag 2006b)	GII/Hu/KR/2007/GII.4[P4]/CBNU2
JX445155	GII.P4 (Den Haag 2006b)	GII/Hu/CA/2006/GII.4[P4]/AlbertaEI438
JX445159	GII.P4 (Den Haag 2006b)	GII/Hu/CA/2008/GII.4[P4]/AlbertaEI009
JX445160	GII.P4 (Den Haag 2006b)	GII/Hu/CA/2008/GII.4[P4]/AlbertaEI102
JX445162	GII.P4 (Den Haag 2006b)	GII/Hu/CA/2008/GII.4[P4]/AlbertaEI425
JX445163	GII.P4 (Den Haag 2006b)	GII/Hu/CA/2009/GII.4[P4]/AlbertaEI109
JX459900	GII.P4 (Den Haag 2006b)	GII/Hu/AU/2011/GII.4[P4]/Randwick/NSW882J
JX459905	GII.P4 (Den Haag 2006b)	GII/Hu/AU/2011/GII.4[P4]/Randwick/NSW938K
JX459906	GII.P4 (Den Haag 2006b)	GII/Hu/AU/2011/GII.4[P4]/Miranda/NSW850K
JX989073	GII.P4 (Den Haag 2006b)	GII/Hu/CN/2010/GII.4[P4]/GZ2010-L26/Guangzhou
KC175343	GII.P4 (Den Haag 2006b)	GII/Hu/VN/2009/GII.4[P4]/Norwalk/10051
KC175345	GII.P4 (Den Haag 2006b)	GII/Hu/VN/2009/GII.4[P4]/Norwalk/10062
KC175348	GII.P4 (Den Haag 2006b)	GII/Hu/VN/2009/GII.4[P4]/Norwalk/10078
KC175357	GII.P4 (Den Haag 2006b)	GII/Hu/VN/2009/GII.4[P4]/Norwalk/10148
KC175360	GII.P4 (Den Haag 2006b)	GII/Hu/VN/2009/GII.4[P4]/Norwalk/10162
KC175370	GII.P4 (Den Haag 2006b)	GII/Hu/VN/2009/GII.4[P4]/Norwalk/10199
KC175372	GII.P4 (Den Haag 2006b)	GII/Hu/VN/2009/GII.4[P4]/Norwalk/10204
KC175373	GII.P4 (Den Haag 2006b)	GII/Hu/VN/2009/GII.4[P4]/Norwalk/10222
KC175374	GII.P4 (Den Haag 2006b)	GII/Hu/VN/2009/GII.4[P4]/Norwalk/10223
KC175376	GII.P4 (Den Haag 2006b)	GII/Hu/VN/2009/GII.4[P4]/Norwalk/10236
KC175377	GII.P4 (Den Haag 2006b)	GII/Hu/VN/2009/GII.4[P4]/Norwalk/10238
KC175379	GII.P4 (Den Haag 2006b)	GII/Hu/VN/2009/GII.4[P4]/Norwalk/10255
KC175381	GII.P4 (Den Haag 2006b)	GII/Hu/VN/2010/GII.4[P4]/Norwalk/10296
KC175390	GII.P4 (Den Haag 2006b)	GII/Hu/VN/2009/GII.4[P4]/Norwalk/20014
KC175392	GII.P4 (Den Haag 2006b)	GII/Hu/VN/2009/GII.4[P4]/Norwalk/20019
KC175396	GII.P4 (Den Haag 2006b)	GII/Hu/VN/2009/GII.4[P4]/Norwalk/20047
KC175397	GII.P4 (Den Haag 2006b)	GII/Hu/VN/2009/GII.4[P4]/Norwalk/20066
KC175402	GII.P4 (Den Haag 2006b)	GII/Hu/VN/2009/GII.4[P4]/Norwalk/20094
KC175404	GII.P4 (Den Haag 2006b)	GII/Hu/VN/2009/GII.4[P4]/Norwalk/20122
KC175410	GII.P4 (Den Haag 2006b)	GII/Hu/VN/2009/GII.4[P4]/Norwalk/20142
KC409239	GII.P4 (Den Haag 2006b)	GII/Hu/VN/2009/GII.4[P4]/10127
KC409262	GII.P4 (Den Haag 2006b)	GII/Hu/VN/2009/GII.4[P4]/20172
KC409263	GII.P4 (Den Haag 2006b)	GII/Hu/VN/2009/GII.4[P4]/20173
KC409265	GII.P4 (Den Haag 2006b)	GII/Hu/VN/2009/GII.4[P4]/20180
KC409271	GII.P4 (Den Haag 2006b)	GII/Hu/VN/2009/GII.4[P4]/20190
KC409273	GII.P4 (Den Haag 2006b)	GII/Hu/VN/2009/GII.4[P4]/20196
KC409274	GII.P4 (Den Haag 2006b)	GII/Hu/VN/2009/GII.4[P4]/20198
KC409278	GII.P4 (Den Haag 2006b)	GII/Hu/VN/2009/GII.4[P4]/20208
KC409279	GII.P4 (Den Haag 2006b)	GII/Hu/VN/2009/GII.4[P4]/20215
KC409280	GII.P4 (Den Haag 2006b)	GII/Hu/VN/2009/GII.4[P4]/20217
KC409282	GII.P4 (Den Haag 2006b)	GII/Hu/VN/2009/GII.4[P4]/20230
KC409286	GII.P4 (Den Haag 2006b)	GII/Hu/VN/2009/GII.4[P4]/20263
KC409289	GII.P4 (Den Haag 2006b)	GII/Hu/VN/2009/GII.4[P4]/20302
KC409290	GII.P4 (Den Haag 2006b)	GII/Hu/VN/2009/GII.4[P4]/20344
KC409293	GII.P4 (Den Haag 2006b)	GII/Hu/VN/2010/GII.4[P4]/20365
KC409294	GII.P4 (Den Haag 2006b)	GII/Hu/VN/2010/GII.4[P4]/20373
KC409295	GII.P4 (Den Haag 2006b)	GII/Hu/VN/2010/GII.4[P4]/20407
KC409297	GII.P4 (Den Haag 2006b)	GII/Hu/VN/2010/GII.4[P4]/20424
KC409313	GII.P4 (Den Haag 2006b)	GII/Hu/VN/2009/GII.4[P4]/30206
KC409317	GII.P4 (Den Haag 2006b)	GII/Hu/VN/2009/GII.4[P4]/30257
KC517365	GII.P4 (Den Haag 2006b)	GII/Hu/TW/2012/GII.4[P4]/Taoyuan/CGMH55
KC517368	GII.P4 (Den Haag 2006b)	GII/Hu/TW/2012/GII.4[P4]/New Taipei/CGMH58
KC517369	GII.P4 (Den Haag 2006b)	GII/Hu/TW/2012/GII.4[P4]/Taoyuan/CGMH59
KC517372	GII.P4 (Den Haag 2006b)	GII/Hu/TW/2012/GII.4[P4]/New Taipei/CGMH62
KC576909	GII.P4 (Den Haag 2006b)	GII/Hu/US/2011/GII.4[P4]/NIHIC4.2
KC894943	GII.P4 (Den Haag 2006b)	GII/Hu/CN/2011/GII.4[P4]/Guangzhou/GZ2010-L91
KC960611	GII.P4 (Den Haag 2006b)	GII/Hu/VN/2010/GII.4[P4]/20384
KC960614	GII.P4 (Den Haag 2006b)	GII/Hu/VN/2009/GII.4[P4]/30116
KC960615	GII.P4 (Den Haag 2006b)	GII/Hu/VN/2009/GII.4[P4]/20119
KF712495	GII.P4 (Den Haag 2006b)	GII/Hu/US/2012/GII.4[P4]/NIHIC17.3

Table S1 (continued). The strains used in this study.

GenBank accession No.	ORF1 genotype	Name
KF712501	GII.P4 (Den Haag 2006b)	GII/Hu/US/2012/GII.4[P4]/NIHIC17.6
KF712502	GII.P4 (Den Haag 2006b)	GII/Hu/US/2013/GII.4[P4]/NIHIC35
KF712510	GII.P4 (Den Haag 2006b)	GII/Hu/US/2010/GII.4[P4]/NIHIC2.2
KJ196287	GII.P4 (Den Haag 2006b)	GII/Hu/JP/2010/GII.4[P4]/Shimada/ASC96
KJ541743	GII.P4 (Den Haag 2006b)	GII/Hu/KR/2011/GII.4[P4]/SGU-110421
KM198489	GII.P4 (Den Haag 2006b)	GII/Hu/VN/2009/GII.4[P4]/10101
KM198494	GII.P4 (Den Haag 2006b)	GII/Hu/VN/2009/GII.4[P4]/10247
KM198515	GII.P4 (Den Haag 2006b)	GII/Hu/VN/2011/GII.4[P4]/C2H-31
KM198517	GII.P4 (Den Haag 2006b)	GII/Hu/VN/2010/GII.4[P4]/30399
KM198532	GII.P4 (Den Haag 2006b)	GII/Hu/VN/2009/GII.4[P4]/10108
KM198545	GII.P4 (Den Haag 2006b)	GII/Hu/VN/2009/GII.4[P4]/10195
KM198564	GII.P4 (Den Haag 2006b)	GII/Hu/VN/2011/GII.4[P4]/C2H-44
KM198570	GII.P4 (Den Haag 2006b)	GII/Hu/VN/2009/GII.4[P4]/20146
KM198574	GII.P4 (Den Haag 2006b)	GII/Hu/VN/2009/GII.4[P4]/10183
KM198575	GII.P4 (Den Haag 2006b)	GII/Hu/VN/2009/GII.4[P4]/20069
KM198576	GII.P4 (Den Haag 2006b)	GII/Hu/VN/2011/GII.4[P4]/C2H-43
KM198578	GII.P4 (Den Haag 2006b)	GII/Hu/VN/2011/GII.4[P4]/C2H-52
KM198579	GII.P4 (Den Haag 2006b)	GII/Hu/VN/2009/GII.4[P4]/10160
KM198584	GII.P4 (Den Haag 2006b)	GII/Hu/VN/2009/GII.4[P4]/10131
KM198587	GII.P4 (Den Haag 2006b)	GII/Hu/VN/2009/GII.4[P4]/20153
KM198588	GII.P4 (Den Haag 2006b)	GII/Hu/VN/2009/GII.4[P4]/10037
KT152148	GII.P4 (Den Haag 2006b)	GII/Hu/US/2006/GII.4[P4]/Minerva
KU311162	GII.P4 (Den Haag 2006b)	GII/Hu/CA/2012/GII.4[P4]/AlbertaSP1
MF140652	GII.P4 (Den Haag 2006b)	GII/Hu/NL/2008/GII.4[P4]/Rotterdam/E7800015
MF140654	GII.P4 (Den Haag 2006b)	GII/Hu/NL/2008/GII.4[P4]/Rotterdam/E7800023
MF140681	GII.P4 (Den Haag 2006b)	GII/Hu/NL/2011/GII.4[P4]/Rotterdam/E1300319
MF140683	GII.P4 (Den Haag 2006b)	GII/Hu/NL/2012/GII.4[P4]/Rotterdam/E1300321
MG049692	GII.P4 (Den Haag 2006b)	GII/Hu/TW/2009/GII.4[P4]/YJB1
AY587988	GII.P4 (Farmington Hills 2002)	GII/Hu/UK/2002/GII.4[P4]/Oxford/B4S2
AY587989	GII.P4 (Farmington Hills 2002)	GII/Hu/UK/2002/GII.4[P4]/Oxford/B2S16
DQ415279	GII.P4 (Farmington Hills 2002)	GII/Hu/IE/2002/GII.4[P4]/Carlow
DQ658413	GII.P4 (Farmington Hills 2002)	GII/Hu/US/2004/GII.4[P4]/MD-2004
JQ798158	GII.P4 (Farmington Hills 2002)	GII/Hu/US/2004/GII.4[P4]/5M
JX126912	GII.P4 (Farmington Hills 2002)	GII/Hu/US/2012/GII.4[P4]/Ohio/7
JX126913	GII.P4 (Farmington Hills 2002)	GII/Hu/US/2012/GII.4[P4]/Ohio/7G
JX445152	GII.P4 (Farmington Hills 2002)	GII/Hu/CA/2004/GII.4[P4]/AlbertaEI131
EF202568	GII.P4 (Hunter_2004)	GII/Hu/CA/2005/GII.4[P4]/Tronto/SK
JX445153	GII.P4 (Hunter_2004)	GII/Hu/CA/2006/GII.4[P4]/AlbertaEI142
MH413070	GII.P4 (Hunter_2004)	GII/Hu/US/2008/GII.4[P4]/0859
AB933738	GII.P4 (New Orleans 2009)	GII/Hu/JP/2009/GII.4[P4]/Niigata5
AB933740	GII.P4 (New Orleans 2009)	GII/Hu/JP/2009/GII.4[P4]/Chiba2
AB933741	GII.P4 (New Orleans 2009)	GII/Hu/JP/2009/GII.4[P4]/Chiba4
AB933742	GII.P4 (New Orleans 2009)	GII/Hu/JP/2009/GII.4[P4]/Nagano2
AB933746	GII.P4 (New Orleans 2009)	GII/Hu/JP/2009/GII.4[P4]/Osaka1
AB933747	GII.P4 (New Orleans 2009)	GII/Hu/JP/2009/GII.4[P4]/Osaka5
AB933748	GII.P4 (New Orleans 2009)	GII/Hu/JP/2011/GII.4[P4]/Ehime5
AB933751	GII.P4 (New Orleans 2009)	GII/Hu/JP/2009/GII.4[P4]/Hiroshima5
AB933753	GII.P4 (New Orleans 2009)	GII/Hu/JP/2009/GII.4[P4]/Ehime4
AB933754	GII.P4 (New Orleans 2009)	GII/Hu/JP/2009/GII.4[P4]/Saga1
AB933755	GII.P4 (New Orleans 2009)	GII/Hu/JP/2009/GII.4[P4]/Saga2
AB933756	GII.P4 (New Orleans 2009)	GII/Hu/JP/2009/GII.4[P4]/Saga3
AB933758	GII.P4 (New Orleans 2009)	GII/Hu/JP/2011/GII.4[P4]/Chiba6
AB933762	GII.P4 (New Orleans 2009)	GII/Hu/JP/2011/GII.4[P4]/Hokkaido4
AB933763	GII.P4 (New Orleans 2009)	GII/Hu/JP/2011/GII.4[P4]/Osaka2
AB933765	GII.P4 (New Orleans 2009)	GII/Hu/JP/2011/GII.4[P4]/Aichi5
AB933767	GII.P4 (New Orleans 2009)	GII/Hu/JP/2011/GII.4[P4]/Hokkaido3
AB933769	GII.P4 (New Orleans 2009)	GII/Hu/JP/2011/GII.4[P4]/Nagano4
AB933771	GII.P4 (New Orleans 2009)	GII/Hu/JP/2011/GII.4[P4]/Hiroshimacity1
GQ845367	GII.P4 (New Orleans 2009)	GII/Hu/AU/2008/GII.4[P4]/Orange/NSW001P

Table S1 (continued). The strains used in this study.

GenBank accession No.	ORF1 genotype	Name
HF952120	GII.P4 (New Orleans 2009)	GII/Hu/UK/2011/GII.4[P4]/00007876
HF952130	GII.P4 (New Orleans 2009)	GII/Hu/UK/2011/GII.4[P4]/00007918
HF952132	GII.P4 (New Orleans 2009)	GII/Hu/UK/2011/GII.4[P4]/00007928
HF952134	GII.P4 (New Orleans 2009)	GII/Hu/UK/2011/GII.4[P4]/00007941
HF952135	GII.P4 (New Orleans 2009)	GII/Hu/UK/2011/GII.4[P4]/00007952
JN400621	GII.P4 (New Orleans 2009)	GII/Hu/TW/2010/GII.4[P4]/CGMH23
JN400622	GII.P4 (New Orleans 2009)	GII/Hu/TW/2010/GII.4[P4]/CGMH24
JN400624	GII.P4 (New Orleans 2009)	GII/Hu/TW/2010/GII.4[P4]/CGMH26
JN595867	GII.P4 (New Orleans 2009)	GII/Hu/US/2010/GII.4[P4]/New Orleans
JQ613552	GII.P4 (New Orleans 2009)	GII/Hu/AU/2010/GII.4[P4]/NSW123B
JQ613570	GII.P4 (New Orleans 2009)	GII/Hu/AU/2009/GII.4[P4]/Rockdale/NSW006D
JQ613571	GII.P4 (New Orleans 2009)	GII/Hu/AU/2010/GII.4[P4]/Miranda/NSW817L
JQ613573	GII.P4 (New Orleans 2009)	GII/Hu/AU/2010/GII.4[P4]/Helensburgh/NSW295E
JX445165	GII.P4 (New Orleans 2009)	GII/Hu/CA/2010/GII.4[P4]/AlbertaEI119
JX445166	GII.P4 (New Orleans 2009)	GII/Hu/CA/2010/GII.4[P4]/AlbertaEI204
JX445168	GII.P4 (New Orleans 2009)	GII/Hu/CA/2011/GII.4[P4]/AlbertaEI388
JX445169	GII.P4 (New Orleans 2009)	GII/Hu/CA/2012/GII.4[P4]/AlbertaEI003
JX448566	GII.P4 (New Orleans 2009)	GII/Hu/KR/2010/GII.4[P4]/Seoul/1071
JX459901	GII.P4 (New Orleans 2009)	GII/Hu/AU/2011/GII.4[P4]/Caringbah/NSW409G
JX846928	GII.P4 (New Orleans 2009)	GII/Hu/US/2011/GII.4[P4]/NIHIC9
JX989074	GII.P4 (New Orleans 2009)	GII/Hu/CN/2011/GII.4[P4]/GZ2010-L87/Guangzhou
KC409240	GII.P4 (New Orleans 2009)	GII/Hu/VN/2010/GII.4[P4]/10370
KC409243	GII.P4 (New Orleans 2009)	GII/Hu/VN/2010/GII.4[P4]/10411
KC409301	GII.P4 (New Orleans 2009)	GII/Hu/VN/2010/GII.4[P4]/20469
KC409302	GII.P4 (New Orleans 2009)	GII/Hu/VN/2010/GII.4[P4]/20477
KC409303	GII.P4 (New Orleans 2009)	GII/Hu/VN/2010/GII.4[P4]/20478
KC463910	GII.P4 (New Orleans 2009)	GII/Hu/US/2012/GII.4[P4]/Ohio/684
KC577174	GII.P4 (New Orleans 2009)	GII/Hu/CN/2011/GII.4[P4]/Jiangsu1
KC960616	GII.P4 (New Orleans 2009)	GII/Hu/VN/2010/GII.4[P4]/20406
KC962462	GII.P4 (New Orleans 2009)	GII/Hu/ZA/2011/GII.4[P4]/Empangeni/8491
KF429766	GII.P4 (New Orleans 2009)	GII/Hu/US/2011/GII.4[P4]/NIHIC13
KF429778	GII.P4 (New Orleans 2009)	GII/Hu/US/2012/GII.4[P4]/NIHIC18.1
KF429788	GII.P4 (New Orleans 2009)	GII/Hu/US/2012/GII.4[P4]/NIHIC20
KF509947	GII.P4 (New Orleans 2009)	GII/Hu/CA/2011/GII.4[P4]/AlbertaEI337
KF712497	GII.P4 (New Orleans 2009)	GII/Hu/US/2012/GII.4[P4]/NIHIC27.2
KJ407073	GII.P4 (New Orleans 2009)	GII/Hu/US/2012/GII.4[P4]/HS292
KJ407075	GII.P4 (New Orleans 2009)	GII/Hu/US/2012/GII.4[P4]/HS288
KJ685402	GII.P4 (New Orleans 2009)	GII/Hu/BD/2012/GII.4[P4]/BG1C0434
KJ685403	GII.P4 (New Orleans 2009)	GII/Hu/BD/2011/GII.4[P4]/BG1C0204
KJ685405	GII.P4 (New Orleans 2009)	GII/Hu/BD/2011/GII.4[P4]/BG1C0282
KJ685408	GII.P4 (New Orleans 2009)	GII/Hu/BD/2011/GII.4[P4]/BG1C0066
KJ685414	GII.P4 (New Orleans 2009)	GII/Hu/BD/2010/GII.4[P4]/BG1C0004
KJ685417	GII.P4 (New Orleans 2009)	GII/Hu/BD/2011/GII.4[P4]/BG1C0270
KJ710245	GII.P4 (New Orleans 2009)	GII/Hu/ZA/2011/GII.4[P4]/CapeTown/6745
KM198488	GII.P4 (New Orleans 2009)	GII/Hu/VN/2010/GII.4[P4]/10325
KM198491	GII.P4 (New Orleans 2009)	GII/Hu/VN/2009/GII.4[P4]/30351
KM198507	GII.P4 (New Orleans 2009)	GII/Hu/VN/2010/GII.4[P4]/C2007
KM198516	GII.P4 (New Orleans 2009)	GII/Hu/VN/2010/GII.4[P4]/10285
KM198544	GII.P4 (New Orleans 2009)	GII/Hu/VN/2010/GII.4[P4]/C2418
KM258130	GII.P4 (New Orleans 2009)	GII/Hu/TW/2012/GII.4[P4]/12-AY-1
KP784691	GII.P4 (New Orleans 2009)	GII/Hu/ZA/2009/GII.4[P4]/Johannesburg/4175
KP784692	GII.P4 (New Orleans 2009)	GII/Hu/ZA/2011/GII.4[P4]/Johannesburg/7028
KP784693	GII.P4 (New Orleans 2009)	GII/Hu/ZA/2011/GII.4[P4]/Empangeni/8501
KP784694	GII.P4 (New Orleans 2009)	GII/Hu/ZA/2012/GII.4[P4]/ Empangeni/8604
KP784695	GII.P4 (New Orleans 2009)	GII/Hu/ZA/2012/GII.4[P4]/ Empangeni/9693
KP784698	GII.P4 (New Orleans 2009)	GII/Hu/ZA/2012/GII.4[P4]/ Johannesburg/BW
KY905331	GII.P4 (New Orleans 2009)	GII/Hu/AU/2016/GII.4[P4]/NSW789Z
KY947546	GII.P4 (New Orleans 2009)	GII/Hu/US/2015/GII.4[P4]/Titusville7426
MF140641	GII.P4 (New Orleans 2009)	GII/Hu/NL/2013/GII.4[P4]/Rotterdam/E7800009
MF140643	GII.P4 (New Orleans 2009)	GII/Hu/NL/2013/GII.4[P4]/Rotterdam/E1300307

Table S1 (continued). The strains used in this study.

GenBank accession No.	ORF1 genotype	Name
MF140644	GII.P4 (New Orleans 2009)	GII/Hu/NL/2014/GII.4[P4]/Rotterdam/E7800018
MF140649	GII.P4 (New Orleans 2009)	GII/Hu/NL/2010/GII.4[P4]/Rotterdam/E7800012
MF140669	GII.P4 (New Orleans 2009)	GII/Hu/NL/2012/GII.4[P4]/Rotterdam/E1300296
MF140670	GII.P4 (New Orleans 2009)	GII/Hu/NL/2013/GII.4[P4]/Rotterdam/E1300297
MF140690	GII.P4 (New Orleans 2009)	GII/Hu/NL/2012/GII.4[P4]/Rotterdam/E7800011
MF140693	GII.P4 (New Orleans 2009)	GII/Hu/NL/2011/GII.4[P4]/Rotterdam/E7800020
MF140695	GII.P4 (New Orleans 2009)	GII/Hu/NL/2013/GII.4[P4]/Rotterdam/E1300281
MF140696	GII.P4 (New Orleans 2009)	GII/Hu/NL/2013/GII.4[P4]/Rotterdam/E1300282
MF140697	GII.P4 (New Orleans 2009)	GII/Hu/NL/2014/GII.4[P4]/Rotterdam/E1300283
MG002632	GII.P4 (New Orleans 2009)	GII/Hu/AU/2017/GII.4[P4]/BNE3
MG002634	GII.P4 (New Orleans 2009)	GII/Hu/AU/2017/GII.4[P4]/BNE5
MH218606	GII.P4 (New Orleans 2009)	GII/Hu/UK/2015/GII.4[P4]/NORO_135_30_01
MH218607	GII.P4 (New Orleans 2009)	GII/Hu/UK/2015/GII.4[P4]/NORO_136_20_02
MH218610	GII.P4 (New Orleans 2009)	GII/Hu/UK/2014/GII.4[P4]/NORO_139_24_10
MH218612	GII.P4 (New Orleans 2009)	GII/Hu/UK/2014/GII.4[P4]/NORO_141_02_11
MH218617	GII.P4 (New Orleans 2009)	GII/Hu/UK/2015/GII.4[P4]/NORO_147_05_01
MH218652	GII.P4 (New Orleans 2009)	GII/Hu/UK/2015/GII.4[P4]/NORO_184_23_09
MH218665	GII.P4 (New Orleans 2009)	GII/Hu/UK/2015/GII.4[P4]/NORO_207_07_12
MH218669	GII.P4 (New Orleans 2009)	GII/Hu/UK/2015/GII.4[P4]/NORO_211_16_12
MH218670	GII.P4 (New Orleans 2009)	GII/Hu/UK/2015/GII.4[P4]/NORO_212_17_12
MH218691	GII.P4 (New Orleans 2009)	GII/Hu/UK/2016/GII.4[P4]/NORO_234_25_01
MH218695	GII.P4 (New Orleans 2009)	GII/Hu/UK/2014/GII.4[P4]/NORO_32_19_06
MH218710	GII.P4 (New Orleans 2009)	GII/Hu/UK/2014/GII.4[P4]/NORO_53_19_07
MH218714	GII.P4 (New Orleans 2009)	GII/Hu/UK/2015/GII.4[P4]/NORO_58-1_13_10
MH413069	GII.P4 (New Orleans 2009)	GII/Hu/US/2010/GII.4[P4]/10149
AY741811	GII.P4 (US95_96)	GII/Hu/GE/1997/GII.4[P4]/Dresden174/pUS-NorII
KJ407076	GII.P4 (US95_96)	GII/Hu/US/2001/GII.4[P4]/HS66
AB447433	GII.P4 (Yerseke_2006a)	GII/Hu/JP/2006/GII.4[P4]/Aomori2
AB447458	GII.P4 (Yerseke_2006a)	GII/Hu/JP/2006/GII.4[P4]/Saga5
AB541267	GII.P4 (Yerseke_2006a)	GII/Hu/JP/2007/GII.4[P4]/Hokkaido5
JX445156	GII.P4 (Yerseke_2006a)	GII/Hu/CA/2008/GII.4[P4]/AlbertaEI498
KC631814	GII.P4 (Yerseke_2006a)	GII/Hu/US/2011/GII.4[P4]/MI001
EF187497	GII.P4	GII/Hu/NZ/2006/GII.4[P4]/Kenepuru/NZ327
EU921344	GII.P4	GII/Hu/IN/2006/GII.4[P4]/Pune/PC15
FJ537136	GII.P4	GII/Hu/US/1988/GII.4[P4]/CHDC3967
JN400599	GII.P4	GII/Hu/TW/2006/GII.4[P4]/CGMH01
JX445154	GII.P4	GII/Hu/CA/2006/GII.4[P4]/AlbertEI190
KC962453	GII.P4	GII/Hu/ZA/2010/GII.4[P4]/Bushbuckridge/5928
KF429787	GII.P4	GII/Hu/US/2012/GII.4[P4]/NIHIC28.5
KF712503	GII.P4	GII/Hu/US/2012/GII.4[P4]/NIHIC28.3
KY947547	GII.P4	GII/Hu/US/2014/GII.4[P4]/Ellsworth 7118
MF140655	GII.P4	GII/Hu/NL/2009/GII.4[P4]/Rotterdam/E7800013
MF140658	GII.P4	GII/Hu/NL/2009/GII.4[P4]/Rotterdam/E1300265
MF140663	GII.P4	GII/Hu/NL/2010/GII.4[P4]/Rotterdam/E7800021
MF140665	GII.P4	GII/Hu/NL/2010/GII.4[P4]/Rotterdam/E1300288
MF140666	GII.P4	GII/Hu/NL/2011/GII.4[P4]/Rotterdam/E1300289
MF140667	GII.P4	GII/Hu/NL/2011/GII.4[P4]/Rotterdam/E1300290
KJ196288	GII.P5	GII/Hu/JP/2002/GII.5[P5]/Saitama/T52
AB039778	GII.P6	GII/Hu/JP/1997/GII.6[P6]/Saitama U16
HQ169542	GII.P6	GII/Hu/US/2005/GII.6[P6]/186
JX989075	GII.P6	GII/Hu/CN/2011/GII.6[P6]/GZ2010-L96/Guangzhou
**KC576910**	**GII.P6**	**GII/Hu/SN/1976/GII.6[P6]/S9c**
KY424346	GII.P6	GII/Hu/US/1971/GII.6[P6]/HenrytonH2
AB039776	GII.P7	GII/Hu/JP/1997/GII.6[P7]/Saitama U3
EF670650	GII.P7	GII/Hu/CN/2006/GII.14[P7]/Shanxi/50106
GU017903	GII.P7	GII/Hu/JP/2008/GII.14[P7]/8533/Maizuru
JX846927	GII.P7	GII/Hu/US/1984/GII.6[P7]/CHDC4073
KJ196278	GII.P7	GII/Hu/JP/2007/GII.14[P7]/Sendai/YG99
KJ196295	GII.P7	GII/Hu/JP/2010/GII.7[P7]/Musashimurayama

The bold letter strain might be estimated the ORF1 genotype in this study.

Table S1 (continued). The strains used in this study.

GenBank accession No.	ORF1 genotype	Name
KJ407072	GII.P7	GII/Hu/US/2010/GII.6[P7]/HS245
KM198498	GII.P7	GII/Hu/VN/2009/GII.6[P7]/20088
KM198519	GII.P7	GII/Hu/VN/2009/GII.6[P7]/30082
KM198531	GII.P7	GII/Hu/VN/2009/GII.6[P7]/30116
KM198534	GII.P7	GII/Hu/VN/2010/GII.6[P7]/30443
KM198549	GII.P7	GII/Hu/VN/2010/GII.6[P7]/30473
KU935739	GII.P7	GII/Hu/CN/2015/GII.6[P7]/0907-26
KX158281	GII.P7	GII/Hu/CN/2015/GII.6[P7]/14-55
KX268709	GII.P7	GII/Hu/US/2014/GII.6[P7]/Maryland
KX752057	GII.P7	GII/Hu/CN/2009/GII.6[P7]/Beijing
KY424344	GII.P7	GII/Hu/US/2012/GII.6[P7]/BethesdaD21
MF140645	GII.P7	GII/Hu/NL/2013/GII.7[P7]/Rotterdam/E1300273
MF140647	GII.P7	GII/Hu/NL/2013/GII.7[P7]/Rotterdam/E1300275
MF140679	GII.P7	GII/Hu/NL/2014/GII.6[P7]/Rotterdam/E1300314
MF140680	GII.P7	GII/Hu/NL/2014/GII.6[P7]/Rotterdam/E1300315
MF802550	GII.P7	GII/Hu/CN/2016/GII.6[P7]/12025
MG557654	GII.P7	GII/Hu/ET/2016/GII.6[P7]/P3
MG571778	GII.P7	GII/Hu/VE/2015/GII.6[P7]/V4B
MG674720	GII.P7	GII/Hu/CN/2017/GII.6[P7]/Fengtai
MH218639	GII.P7	GII/Hu/UK/2014/GII.6[P7]/NORO_170_23_09
MH218640	GII.P7	GII/Hu/UK/2015/GII.6[P7]/NORO_171_09_03
MH218641	GII.P7	GII/Hu/UK/2015/GII.6[P7]/NORO_172_02_04
MH218642	GII.P7	GII/Hu/UK/2015/GII.6[P7]/NORO_173_08_05
MH218643	GII.P7	GII/Hu/UK/2015/GII.6[P7]/NORO_174_03_06
MH218650	GII.P7	GII/Hu/UK/2015/GII.6[P7]/NORO_182_19_09
MH218658	GII.P7	GII/Hu/UK/2015/GII.7[P7]/NORO_197_04_11
MH218661	GII.P7	GII/Hu/UK/2015/GII.7[P7]/NORO_200_25_11
MH218667	GII.P7	GII/Hu/UK/2015/GII.6[P7]/NORO_209_15_12
MH218673	GII.P7	GII/Hu/UK/2015/GII.6[P7]/NORO_215_18_12
MH218687	GII.P7	GII/Hu/UK/2016/GII.6[P7]/NORO_229_12_01
MH218692	GII.P7	GII/Hu/UK/2016/GII.7[P7]/NORO_236_29_01
MH218719	GII.P7	GII/Hu/UK/2015/GII.6[P7]/NORO_68-7_18_03
MH791993	GII.P7	GII/Hu/US/2017/GII.6[P7]/ST368
AB039780	GII.P8	GII/Hu/JP/1998/GII.8[P8]/Saitama U25
JX846926	GII.P8	GII/Hu/US/1988/GII.7[P8]/CHDC3936
AB220921	GII.P12	GII/Hu/JP/2005/GII.4[P12]/Chiba/04-1050
AB220922	GII.P12	GII/Hu/JP/2005/GII.4[P12]/Sakai/04-179
AB447448	GII.P12	GII/Hu/JP/2006/GII.4[P12]/Sakai2
AB933728	GII.P12	GII/Hu/JP/2009/GII.4[P12]/Akita5
AF504671	GII.P12	GII/Hu/VN/2000/GII.10[P12]/026
GU980585	GII.P12	GII/Hu/KR/2006/GII.3[P12]/CBNU
GU991355	GII.P12	GII/Hu/CN/2009/GII.3[P12]/Shanghai/SH312
KF306213	GII.P12	GII/Hu/CN/2013/GII.3[P12]/Jingzhou/2013402
KJ196276	GII.P12	GII/Hu/JP/2002/GII.13[P12]/Saitama/T80
KJ196282	GII.P12	GII/Hu/JP/2001/GII.12[P12]/Saitama/T15
KJ196294	GII.P12	GII/Hu/JP/2000/GII.12[P12]/Saitama/KU16
KY348697	GII.P12	GII/Hu/CN/2013/GII.3[P12]/Guangzhou/GZ2013-L20
KY905334	GII.P12	GII/Hu/AU/2016/GII.3[P12]/QLDB207
LC209435	GII.P12	GII/Hu/JP/2004/GII.2[P12]/Tochigi-92
LC421227	GII.P12	GII/Hu/JP/2017/GII.4[P12]/330077/Tokyo
MH260494	GII.P12	GII/Hu/US/2017/GII.3[P12]/ST173
NC_029646	GII.P12	GII/Hu/JP/1999/GII.12[P12]/Norovirus GII
KC464505	GII.P16	GII/Hu/TW/2011/GII.2[P16]/CGMH47
KF895841	GII.P16	GII/Hu/RU/2012/GII.3[P16]/Smolensk/S12-31
KF944111	GII.P16	GII/Hu/RU/2011/GII.3[P16]/Novosibirsk/Nsk-N1648
KJ196286	GII.P16	GII/Hu/JP/2002/GII.17[P16]/Saitama/T87
KJ407074	GII.P16	GII/Hu/US/2011/GII.2[P16]/HS255
KM036380	GII.P16	GII/Hu/TW/2013/GII.13[P16]/New/Taipei/13-BA-1
KT779557	GII.P16	GII/Hu/RU/2012/GII.3[P16]/Omsk/O1370
KX907727	GII.P16	GII/Hu/US/2015/GII.4[P16]/CA3477

Table S1 (continued). The strains used in this study.

GenBank accession No.	ORF1 genotype	Name
KY771081	GII.P16	GII/Hu/HK/2016/GII.2[P16]/CUHK-NS-1082
KY865306	GII.P16	GII/Hu/US/2016/GII.2[P16]/Santa Rosa 1764
KY865307	GII.P16	GII/Hu/US/2016/GII.2[P16]/Nashville 2122
KY887601	GII.P16	GII/Hu/UK/2016/GII.4[P16]/NOR-2565
KY887602	GII.P16	GII/Hu/UK/2015/GII.4[P16]/NOR-2516
KY887604	GII.P16	GII/Hu/UK/2015/GII.4[P16]/NOR-2518
KY887605	GII.P16	GII/Hu/UK/2015/GII.4[P16]/NOR-2520
KY887606	GII.P16	GII/Hu/UK/2016/GII.3[GII.P16]/NOR-2598
KY905335	GII.P16	GII/Hu/AU/2016/GII.4[P16]/QLDB309
KY905337	GII.P16	GII/Hu/AU/2016/GII.2[P16]/QLDB512
KY947548	GII.P16	GII/Hu/US/2016/GII.13[P16]/Carlsbad 4246
KY947549	GII.P16	GII/Hu/US/2016/GII.4[P16]/CS4243
LC145786	GII.P16	GII/Hu/JP/2012/GII.2[P16]/Akita8
LC145788	GII.P16	GII/Hu/JP/2012/GII.2[P16]/Niigata5
LC145789	GII.P16	GII/Hu/JP/2012/GII.2[P16]/Fukui1
LC145790	GII.P16	GII/Hu/JP/2012/GII.2[P16]/Fukui2
LC145792	GII.P16	GII/Hu/JP/2012/GII.2[P16]/Saitama5
LC145795	GII.P16	GII/Hu/JP/2012/GII.2[P16]/Hiroshimaciy1
LC145803	GII.P16	GII/Hu/JP/2014/GII.2[P16]/Osaka5
LC145808	GII.P16	GII/Hu/JP/2014/GII.2[P16]/Ehime5
LC175468	GII.P16	GII/Hu/JP/2016/GII.4[P16]/Kawasaki194
LC209432	GII.P16	GII/Hu/JP/2012/GII.2[P16]/Tochigi-30
LC209434	GII.P16	GII/Hu/JP/2014/GII.2[P16]/Tochigi-17
LC209441	GII.P16	GII/Hu/JP/2014/GII.2[P16]/Saitama-126
LC209442	GII.P16	GII/Hu/JP/2013/GII.2[P16]/Saitama-125
LC209445	GII.P16	GII/Hu/JP/2012/GII.2[P16]/Saitama-122
LC209446	GII.P16	GII/Hu/JP/2012/GII.2[P16]/Saitama-121
LC209447	GII.P16	GII/Hu/JP/2011/GII.2[P16]/Saitama-51
LC209453	GII.P16	GII/Hu/JP/2011/GII.2[P16]/Osaka-023
LC209454	GII.P16	GII/Hu/JP/2010/GII.2[P16]/Osaka-019
LC209455	GII.P16	GII/Hu/JP/2013/GII.2[P16]/Miyagi-8
LC209458	GII.P16	GII/Hu/JP/2014/GII.2[P16]/Kanagawa-52
LC209459	GII.P16	GII/Hu/JP/2010/GII.2[P16]/Kanagawa-51
LC209461	GII.P16	GII/Hu/JP/2009/GII.2[P16]/Kanagawa-49
LC209466	GII.P16	GII/Hu/JP/2012/GII.2[P16]/Hokkaido-18
LC209470	GII.P16	GII/Hu/JP/2014/GII.2[P16]/Hiroshima-30
LC209475	GII.P16	GII/Hu/JP/2013/GII.2[P16]/Ehime-9
LC209476	GII.P16	GII/Hu/JP/2013/GII.2[P16]/Ehime-8
LC209477	GII.P16	GII/Hu/JP/2013/GII.2[P16]/Ehime-6
LC209478	GII.P16	GII/Hu/JP/2012/GII.2[P16]/Ehime-46
LC209479	GII.P16	GII/Hu/JP/2011/GII.2[P16]/Ehime-45
LC209480	GII.P16	GII/Hu/JP/2010/GII.2[P16]/Ehime-44
MF167650	GII.P16	GII/Hu/CN/2017/GII.2[P16]/JSCZ201703-19
MF802551	GII.P16	GII/Hu/CN/2016/GII.2[P16]/120952/
MG002630	GII.P16	GII/Hu/AU/2017/GII.4[P16]/BNE1
MG002631	GII.P16	GII/Hu/AU/2017/GII.4[P16]/BNE2
MG002633	GII.P16	GII/Hu/AU/2017/GII.4[P16]/BNE4
MG572182	GII.P16	GII/Hu/CN/2017/GII.1[P16]/SDJN170450
MG745985	GII.P16	GII/Hu/CN/2017GII.2[P16]/GZ60
MG745986	GII.P16	GII/Hu/CN/2017GII.2[P16]/GZ72
MG745987	GII.P16	GII/Hu/CN/2017/GII.2[P16]/GZ78
MG745991	GII.P16	GII/Hu/CN/2017/GII.2[P16]/GZ1619
MG745992	GII.P16	GII/Hu/CN/2017/GII.2[P16]/GZ1621
MG745993	GII.P16	GII/Hu/CN/2017/GII.2[P16]/GZ2043
MG745994	GII.P16	GII/Hu/CN/2017/GII.2[P16]/GZ2113
MG745996	GII.P16	GII/Hu/CN/2016/GII.2[P16]/GZ28491
MG745998	GII.P16	GII/Hu/CN/2016/GII.2[P16]/GZ28587
MG745999	GII.P16	GII/Hu/CN/2016/GII.2[P16]/GZ28626
MG746000	GII.P16	GII/Hu/CN/2016/GII.2[P16]/GZ29003
MG746003	GII.P16	GII/Hu/CN/2016/GII.2[P16]/FJ16235606

Table S1 (continued). The strains used in this study.

GenBank accession No.	ORF1 genotype	Name
MG746005	GII.P16	GII/Hu/CN/2017/GII.2[P16]/BJSYL53
MG746006	GII.P16	GII/Hu/CN/2017/GII.2[P16]/BJSYL75
MG746008	GII.P16	GII/Hu/CN/2016/GII.2[P16]/CQ031
MG746009	GII.P16	GII/Hu/CN/2016/GII.2[P16]/CQ035
MG746012	GII.P16	GII/Hu/CN/2017/GII.2[P16]/GX170034-4
MG746015	GII.P16	GII/Hu/CN/2016/GII.2[P16]/GZ27188
MG746017	GII.P16	GII/Hu/CN/2016/GII.2[P16]/HNCD7
MG746018	GII.P16	GII/Hu/CN/2017/GII.2[P16]/JNCH4
MG746021	GII.P16	GII/Hu/CN/2017/GII.2[P16]/JSWXJINYQ
MG746022	GII.P16	GII/Hu/CN/2017/GII.2[P16]/JSYZ25
MG746024	GII.P16	GII/Hu/CN/2017/GII.2[P16]/HNLD01
MG746026	GII.P16	GII/Hu/CN/2017/GII.2[P16]/LNLY502
MG746027	GII.P16	GII/Hu/CN/2016/GII.2[P16]/CQ1
MG746028	GII.P16	GII/Hu/CN/2016/GII.2[P16]/CQ5
MG746029	GII.P16	GII/Hu/CN/2016/GII.2[P16]/SC1
MG746031	GII.P16	GII/Hu/CN/2017/GII.2[P16]/LNSY3
MG746032	GII.P16	GII/Hu/CN/2017/GII.2[P16]/SZ45
MG746033	GII.P16	GII/Hu/CN/2016/GII.2[P16]/SZ123
MG746034	GII.P16	GII/Hu/CN/2017/GII.2[P16]/SZ127
MG746035	GII.P16	GII/Hu/CN/2016/GII.2[P16]/SZ136
MG746037	GII.P16	GII/Hu/CN/2016/GII.2[P16]/SZ205
MG746038	GII.P16	GII/Hu/CN/2016/GII.2[P16]/SZ325
MG746040	GII.P16	GII/Hu/CN/2016/GII.2[P16]/BJHD1608Y123
MG746041	GII.P16	GII/Hu/CN/2017/GII.2[P16]/BJFT359
MG746043	GII.P16	GII/Hu/CN/2017/GII.2[P16]/BJFT463
MG746045	GII.P16	GII/Hu/CN/2017/GII.2[P16]/BJFT694
MH218591	GII.P16	GII/Hu/UK/2014/GII.17[P16]/NORO_121_30_11
MH260478	GII.P16	GII/Hu/US/2017/GII.2[P16]/STMB
MH260479	GII.P16	GII/Hu/US/2017/GII.4[P16]/ST709
MH260482	GII.P16	GII/Hu/US/2017/GII.4[P16]/ST480
MH260483	GII.P16	GII/Hu/US/2017/GII.4[P16]/ST466
MH260489	GII.P16	GII/Hu/US/2017/GII.4[P16]/ST295
MH260491	GII.P16	GII/Hu/US/2017/GII.4[P16]/ST256
MH260499	GII.P16	GII/Hu/US/2017/GII.4[P16]/ST155
MH260501	GII.P16	GII/Hu/US/2017/GII.2[P16]/ST136
MH260506	GII.P16	GII/Hu/US/2017/GII.4[P16]/ST109
MH260508	GII.P16	GII/Hu/US/2017/GII.4[P16]/ST103
MH260509	GII.P16	GII/Hu/US/2017/GII.2[P16]/ST97
MH608287	GII.P16	GII/Hu/CM/2014/GII.13[P16]/CMRHP59
MH791994	GII.P16	GII/Hu/US/2017/GII.4[P16]/ST104
MH979229	GII.P16	GII/Hu/TW/2018/GII.2[P16]/CGMH1420
NC_039476	GII.P16	GII/Hu/CN/2016/GII.2[P16]/BJSMQ
NC_039477	GII.P16	GII/Hu/UK/2016/GII.4[P16]/NorovirusGII
AB983218	GII.P17	GII/Hu/JP/2014/GII.17[P17]/Kawasaki323
KP676383	GII.P17	GII/Hu/CN/2013/GII.17[P17]/Nanjing010141
KP998539	GII.P17	GII/Hu/HK/2014/GII.17[P17]/CUHK-NS-463
KR154230	GII.P17	GII/Hu/TW/2015/GII.17[P17]/CGMH69
KR154231	GII.P17	GII/Hu/TW/2015/GII.17[P17]/CGMH70
KT326180	GII.P17	GII/Hu/HK/2014/GII.17[P17]/CUHK-NS-405
KT326181	GII.P17	GII/Hu/HK/2015/GII.17[P17]/CUHK-NS-649
KT326182	GII.P17	GII/Hu/HK/2015/GII.17[P17]/CUHK-NS-667
KT780394	GII.P17	GII/Hu/HK/2014/GII.17[P17]/CUHK-NS-469
KT780395	GII.P17	GII/Hu/HK/2014/GII.17[P17]/CUHK-NS-482
KT780397	GII.P17	GII/Hu/HK/2014/GII.17[P17]/CUHK-NS-494
KT780401	GII.P17	GII/Hu/HK/2015/GII.17[P17]/CUHK-NS-517
KT780406	GII.P17	GII/Hu/HK/2015/GII.17[P17]/CUHK-NS-600
KT780410	GII.P17	GII/Hu/HK/2015/GII.17[P17]/CUHK-NS-637
KT780413	GII.P17	GII/Hu/HK/2015/GII.17[P17]/CUHK-NS-653
KT780414	GII.P17	GII/Hu/HK/2015/GII.17[P17]/CUHK-NS-655

Table S1 (continued). The strains used in this study.

GenBank accession No.	ORF1 genotype	Name
KT970372	GII.P17	GII/Hu/CN/2015/GII.17[P17]/Guangzhou/GZ2015-L325
KT970375	GII.P17	GII/Hu/CN/2015/GII.17[P17]/Guangzhou/GZ2015-L340
KT970377	GII.P17	GII/Hu/CN/2015/GII.17[P17]/Guangzhou/GZ2015-L362
KT992790	GII.P17	GII/Hu/CN/2015/GII.17[P17]/HKkaohao/Nanyang
KU557783	GII.P17	GII/Hu/CN/2015/GII.17[P17]/35-0584/GD-JM
KU557788	GII.P17	GII/Hu/CN/2013/GII.17[P17]/2238/GD-JM
KU561249	GII.P17	GII/Hu/HK/2015/GII.17[P17]/CUHK-NS-616
KU561251	GII.P17	GII/Hu/KR/2013/GII.17[P17]/CAU-85
KX356908	GII.P17	GII/Hu/CN/2015/GII.17[P17]/KM1509
KY392868	GII.P17	GII/Hu/BR/2015/GII.17[P17]/LVCA_24785
KY424349	GII.P17	GII/Hu/US/2014/GII.17[P17]/GaithersburgD7
KY905332	GII.P17	GII/Hu/AU/2015/GII.17[P17]/NSW543Q
LC043167	GII.P17	GII/Hu/JP/2013/GII.17[P17]/Saitama5203
LC043305	GII.P17	GII/Hu/JP/2014/GII.17[P17]/Nagano8-1
MF918359	GII.P17	GII/Hu/CN/2015/GII.17[P17]/GX213
MH218689	GII.P17	GII/Hu/UK/2016/GII.17[P17]/NORO_231_20_01
EU424333	GII.P20	GII/Hu/DE/2005/GII.20[P20]/Leverkusen267
EU921389	GII.P21	GII/Hu/IN/2007/GII.3[P21]/PC52
KJ196284	GII.P21	GII/Hu/JP/2007/GII.21[P21]/YO284
KM198493	GII.P21	GII/Hu/VN/2010/GII.3[P21]/30468
KM198496	GII.P21	GII/Hu/VN/2010/GII.3[P21]/20419
KM198509	GII.P21	GII/Hu/VN/2010/GII.3[P21]/20479
KM198511	GII.P21	GII/Hu/VN/2011/GII.3[P21]/C2H-24
KM198529	GII.P21	GII/Hu/VN/2011/GII.3[P21]/C2H-27
KM198547	GII.P21	GII/Hu/VN/2011/GII.3[P21]/C2H-47
KM198563	GII.P21	GII/Hu/VN/2010/GII.3[P21]/C2365
KX079488	GII.P21	GII/Hu/KR/2015/GII.21[P21]/JW
MF140684	GII.P21	GII/Hu/NL/2011/GII.3[P21]/E1300322
MF140685	GII.P21	GII/Hu/NL/2011/GII.3[P21]/E1300323
MF140686	GII.P21	GII/Hu/NL/2012/GII.3[P21]/E1300324
MF140687	GII.P21	GII/Hu/NL/2012/GII.3[P21]/E1300325
MF140688	GII.P21	GII/Hu/NL/2012/GII.3[P21]/E1300326
MF140689	GII.P21	GII/Hu/NL/2012/GII.3[P21]/E1300327
MH218573	GII.P21	GII/Hu/UK/2014/GII.3[P21]/NORO_103-1_07_07
MH218575	GII.P21	GII/Hu/UK/2014/GII.3[P21]/NORO_105_05_07
MH218577	GII.P21	GII/Hu/UK/2014/GII.3[P21]/NORO_107_11_07
MH218580	GII.P21	GII/Hu/UK/2014/GII.3[P21]/NORO_110_06_08
MH218583	GII.P21	GII/Hu/UK/2014/GII.3[P21]/NORO_113_04_09
MH218584	GII.P21	GII/Hu/UK/2014/GII.3[P21]/NORO_114_14_09
MH218587	GII.P21	GII/Hu/UK/2014/GII.3[P21]/NORO_117_01_11
MH218589	GII.P21	GII/Hu/UK/2014/GII.3[P21]/NORO_119_27_11
MH218590	GII.P21	GII/Hu/UK/2014/GII.3[P21]/NORO_120_28_11
MH218593	GII.P21	GII/Hu/UK/2015/GII.3[P21]/NORO_123_02_01
MH218594	GII.P21	GII/Hu/UK/2015/GII.3[P21]/NORO_124_10_01
MH218595	GII.P21	GII/Hu/UK/2015/GII.3[P21]/NORO_125_19_01
MH218596	GII.P21	GII/Hu/UK/2015/GII.3[P21]/NORO_126_17_02
MH218597	GII.P21	GII/Hu/UK/2015/GII.3[P21]/NORO_127_05_03
MH218599	GII.P21	GII/Hu/UK/2015/GII.3[P21]/NORO_128_07_03
MH218600	GII.P21	GII/Hu/UK/2015/GII.3[P21]/NORO_129_16_03
MH218601	GII.P21	GII/Hu/UK/2015/GII.3[P21]/NORO_130_25_03
MH218602	GII.P21	GII/Hu/UK/2015/GII.3[P21]/NORO_131_08_04
MH218603	GII.P21	GII/Hu/UK/2015/GII.3[P21]/NORO_132_01_05
MH218618	GII.P21	GII/Hu/UK/2015/GII.3[P21]/NORO_147-2_10_12
MH218630	GII.P21	GII/Hu/UK/2015/GII.3[P21]/NORO_160_14_04
MH218646	GII.P21	GII/Hu/UK/2016/GII.3[P21]/NORO_177-2_26_01
MH218651	GII.P21	GII/Hu/UK/2015/GII.13[P21]/NORO_183_23_09
MH218653	GII.P21	GII/Hu/UK/2015/GII.3[P21]/NORO_185_28_09
MH218654	GII.P21	GII/Hu/UK/2015/GII.3[P21]/NORO_186_28_09
MH218690	GII.P21	GII/Hu/UK/2016/GII.3[P21]/NORO_232_20_01
MH218712	GII.P21	GII/Hu/UK/2014/GII.3[P21]/NORO_55_15_02

Table S1 (continued). The strains used in this study.

GenBank accession No.	ORF1 genotype	Name
MH218732	GII.P21	GII/Hu/UK/2014/GII.3[P21]/NORO_93_03_12
MG495081	GII.P24	GII/Hu/PE/2014/GII.24[P24]/Loreto6424
MG495084	GII.P24	GII/Hu/US/2013/GII.24[P24]/EdenPrairie5457
MG495083	GII.P25	GII/Hu/BD/2012/GII.25[P25]/Dhaka1928
AY134748	GII.P30	GII/Hu/US/1976/GII.2[P30]/Snow Mountain
JX846925	GII.P30	GII/Hu/MY/1978/GII.2[P30]/KL109
KC597138	GII.P30	GII/Hu/US/1975/GII.2[P30]/CHDC2596
KF429769	GII.P30	GII/Hu/US/1975/GII.2[P30]/SnowMountRS
AB541319	GII.P31	GII/Hu/JP/2007/GII.4[P31]/Osaka1
AB541321	GII.P31	GII/Hu/JP/2007/GII.4[P31]/Osaka2
EU921388	GII.P31	GII/Hu/IN/2007/GII.4[P31]/PC51
GQ845369	GII.P31	GII/Hu/AU/2008/GII.4[P31]/NSW390I
JX459907	GII.P31	GII/Hu/AU/2012/GII.4[P31]/NSW3309
JX459908	GII.P31	GII/Hu/AU/2012/GII.4[P31]/NSW0514
KC175323	GII.P31	GII/Hu/HK/2012/GII.4[P31]/CUHK3630
KC517361	GII.P31	GII/Hu/TW/2012/GII.4[P31]/CGMH51
KC517362	GII.P31	GII/Hu/TW/2012/GII.4[P31]/CGMH52
KC517364	GII.P31	GII/Hu/TW/2012/GII.4[P31]/CGMH54
KC517376	GII.P31	GII/Hu/TW/2012/GII.4[P31]/CGMH66
KC517377	GII.P31	GII/Hu/TW/2012/GII.4[P31]/CGMH67
KC517378	GII.P31	GII/Hu/TW/2012/GII.4[P31]/CGMH68
KC577175	GII.P31	GII/Hu/CN/2012/GII.4[P31]/Jiangsu2
KC631827	GII.P31	GII/Hu/HK/2012/GII.4[P31]/CUHK6080
KF306214	GII.P31	GII/Hu/CN/2013/GII.4[P31]/Jingzhou/2013403
KF509946	GII.P31	GII/Hu/CA/2012/GII.4[P31]/AlbertaEI063
KJ196281	GII.P31	GII/Hu/JP/2012/GII.4[P31]/Fukuyama/5
KJ196293	GII.P31	GII/Hu/JP/2012/GII.4[P31]/Fukuyama/1
KJ196296	GII.P31	GII/Hu/TW/2012/GII.4[P31]/Taipei/105
KJ649705	GII.P31	GII/Hu/HK/2013/GII.4[P31]/CUHK-NS-141
KJ685412	GII.P31	GII/Hu/BG/2012/GII.4[P31]/BG1C0405
KJ955493	GII.P31	GII/Hu/CN/2012/GII.4[P31]/Nanshan/OB/12
KM272334	GII.P31	GII/Hu/KR/2012/GII.4[P31]/gg-12-08-04
KP784696	GII.P31	GII/Hu/ZA/2012/GII.4[P31]/CapeTown/9772
KP784697	GII.P31	GII/Hu/ZA/2012/GII.4[P31]/CapeTown/10105
KT202793	GII.P31	GII/Hu/CN/2013/GII.4[P31]/Guangzhou/GZ2013-L10
KT202794	GII.P31	GII/Hu/CN/2014/GII.4[P31]/Guangzhou/GZ2014-L106
KT202796	GII.P31	GII/Hu/CN/2014/GII.4[P31]/Guangzhou/GZ2014-L132
KT202797	GII.P31	GII/Hu/CN/2014/GII.4[P31]/Guangzhou/GZ2014-L295
KT202798	GII.P31	GII/Hu/CN/2014/GII.4[P31]/Guangzhou/GZ2014-L307
KT589391	GII.P31	GII/Hu/HK/2015/GII.17[P31]/CUHK-NS-682
KU311158	GII.P31	GII/Hu/CA/2014/GII.4[P31]/AlbertaEI350
KX158279	GII.P31	GII/Hu/CA/2015/GII.4[P31]/13-38
KX158283	GII.P31	GII/Hu/CA/2015/GII.4[P31]/15-58
KX586330	GII.P31	GII/Hu/CN/2015/GII.4[P31]/ZJ01
KY421039	GII.P31	GII/Hu/US/2015/GII.4[P31]/Variant2015
KY424331	GII.P31	GII/Hu/US/2012/GII.4[P31]/RockvilleD21
KY486271	GII.P31	GII/Hu/US/2013/GII.4[P31]/Sydney
KY905333	GII.P31	GII/Hu/AU/2016/GII.4[P31]/QLDB101
LC066046	GII.P31	GII/Hu/JP/2015/GII.4[P31]/Osaka/OSF78
LC209439	GII.P31	GII/Hu/JP/2014/GII.2[P31]/Saitama-127
MF140636	GII.P31	GII/Hu/NL/2013/GII.4[P31]/Rotterdam/E78000008
MF140637	GII.P31	GII/Hu/NL/2013/GII.4[P31]/Rotterdam/E1300285
MF140639	GII.P31	GII/Hu/NL/2014/GII.4[P31]/Rotterdam/E1300287
MF140640	GII.P31	GII/Hu/NL/2014/GII.4[P31]/Rotterdam/E7800017
MF140674	GII.P31	GII/Hu/NL/2013/GII.4[P31]/Rotterdam/E1300308
MG214988	GII.P31	GII/Hu/CN/2017/GII.4[P31]/Jinan/JN010
MG557655	GII.P31	GII/Hu/ET/2016/GII.10[P31]/P4
MG786781	GII.P31	GII/Hu/TH/2015/GII.4[P31]/DBM15-156
MH218605	GII.P31	GII/Hu/UK/2015/GII.4[P31]/NORO_134_07_02
MH218614	GII.P31	GII/Hu/UK/2014/GII.4[P31]/NORO_143_12_11

Table S1 (continued). The strains used in this study.

GenBank accession No.	ORF1 genotype	Name
MH218615	GII.P31	GII/Hu/UK/2014/GII.4[P31]/NORO_144_13_11
MH218623	GII.P31	GII/Hu/UK/2015/GII.4[P31]/NORO_152_02_02
MH218624	GII.P31	GII/Hu/UK/2015/GII.4[P31]/NORO_153_19_02
MH218625	GII.P31	GII/Hu/UK/2015/GII.4[P31]/NORO_154_02_03
MH218626	GII.P31	GII/Hu/UK/2015/GII.4[P31]/NORO_155_02_03
MH218627	GII.P31	GII/Hu/UK/2015/GII.4[P31]/NORO_156_02_03
MH218629	GII.P31	GII/Hu/UK/2015/GII.4[P31]/NORO_158_01_04
MH218631	GII.P31	GII/Hu/UK/2015/GII.4[P31]/NORO_161_27_04
MH218632	GII.P31	GII/Hu/UK/2015/GII.4[P31]/NORO_162-1_03_05
MH218637	GII.P31	GII/Hu/UK/2015/GII.4[P31]/NORO_168_23_06
MH218648	GII.P31	GII/Hu/UK/2015/GII.2[P31]/NORO_180_01_09
MH218657	GII.P31	GII/Hu/UK/2015/GII.2[P31]/NORO_196_18_10
MH218663	GII.P31	GII/Hu/UK/2015/GII.4[P31]/NORO_205_02_12
MH218697	GII.P31	GII/Hu/UK/2015/GII.2[P31]/NORO_34-7_30_10
MH218701	GII.P31	GII/Hu/UK/2014/GII.4[P31]/NORO_42_01_09
MH218706	GII.P31	GII/Hu/UK/2014/GII.4[P31]/NORO_48-1_01_07
MH218707	GII.P31	GII/Hu/UK/2014/GII.4[P31]/NORO_49_15_10
MH218708	GII.P31	GII/Hu/UK/2014/GII.4[P31]/NORO_50_30_07
MH218711	GII.P31	GII/Hu/UK/2014/GII.4[P31]/NORO_54-1_08_09
MH218733	GII.P31	GII/Hu/UK/2014/GII.2[P31]/NORO_94_23_10
MH979230	GII.P31	GII/Hu/TW/2018/GII.4[P31]/CGMH1411
MF405169	GII.P32	GII/Hu/US/1971/GII.2[P32]/HenrytonSP17
GQ845370	GII.P33	GII/Hu/AU/2008/GII.12[P33]/StGeorge/NSW199U
HQ449728	GII.P33	GII/Hu/US/2010/GII.12[P33]/HS210
HQ664990	GII.P33	GII/Hu/US/2010/GII.12[P33]/HS206
JQ613568	GII.P33	GII/Hu/AU/2009/GII.12[P33]/Wahroonga/NSW004P
KC464499	GII.P33	GII/Hu/TW/2010/GII.12[P33]/CGMH41
KC597145	GII.P33	GII/Hu/US/2010/GII.12[P33]/NIHIC6
MF668937	GII.P33	GII/Hu/ID/2015/GII.1[P33]/ITD11-3
MH218731	GII.P33	GII/Hu/UK/2015/GII.1[P33]/NORO_92_31_01
KC576911	GII.P35	GII/Hu/CF/1977/GII.P35/B17
KJ194507	GII.P37	GII/Hu/NL/1995/GII.1[P37]/Amsterdam/3
AB684675	GII.P39	GII/Hu/JP/1975/GII.4[P39]/21-5/Tokyo
FJ537134	GII.P39	GII/Hu/US/1974/GII.4[P39]/CHDC5191
FJ537138	GII.P39	GII/Hu/US/1977/GII.4[P39]/CHDC4871
KC576915	GII.P39	GII/Hu/MY/1978/GII.4[P39]/KL45
DQ366347	GII.P40	GII/Hu/JP/2004/GII.2[P40]/OsakaNI
KJ196277	GII.P40	GII/Hu/JP/2001/GII.5[P40]/Saitama/T49
KM036379	GII.P40	GII/Hu/TW/2012/GII.5[P40]/Taoyuan/12-BB-4
KU311160	GII.P40	GII/Hu/CA/2013/GII.5[P40]/AlbertaEI390
MF802553	GII.P40	GII/Hu/CN/2016/GII.5[P40]/12196/16
JX846924	GII.P41	GII/Hu/HK/1978/GII.3[P41]/HK71
KC597144	GII.P41	GII/Hu/HK/1977/GII.3[P41]/HK46
KY442319	GII.P41	GII/Hu/US/1972/GII.3[P41]/ShippensburgB24
MG495082	GII.PNA5	GII/Hu/BD/2012/GII.22[PNA5]/Dhaka1940
MG557653	GII.PNA7	GII/Hu/ET/2016/GII.PNA7/P8
M87661	GI.P1	GI/Hu/US/1968/GI.1[P1]/Norwalk
HQ392821	GII.P11	GII/Po/CN/2009/GII.11[P11]/Ch6
AY823305	GII.P18	GII/Po/US/2003/GII.18[P18]/OH-QW125
AJ011099	GIII.P1	GIII/Bo/DE/1980/GIII.P1/Jena
NC029647	GIV.P1	GIV/Hu/AU/2010/GIV.P1/LakeMacquarie/NSW2680

Table S2. Statistical analyses with the multiple comparisons for phylogenetic distance in the present NoV GII Pro strains.

	GII.P2	GII.P4	GII.P7	GII.P12	GII.P16	GII.P17	GII.P21	GII.P31
GII.P2		4.5E-3	4.7E-12	1.4E-4	7.8E-1	< 2E-16	2.1E-1	1.2E-5
GII.P4	**		< 2E-16	4.2E-2	2.7E-3	< 2E-16	< 2E-16	< 2E-16
GII.P7	***	***		< 2E-16	< 2E-16	< 2E-16	< 2E-16	< 2E-16
GII.P12	***	*	***		2.1E-1	< 2E-16	6.1E-13	< 2E-16
GII.P16	-	**	***	-		< 2E-16	1.5E-6	< 2E-16
GII.P17	***	***	***	***	***		< 2E-16	< 2E-16
GII.P21	-	***	***	***	***	***		< 2E-16
GII.P31	***	***	***	***	***	***	***	

The *p*-values and significant signs were shown in the upper and lower sides, respectively.

*$p < 0.05$, ** $p < 0.01$, ***: $p < 0.001$,   -: no significant difference

Table S3. The substitution sites and the negative selection sites of NoV GII strains.

Lineage	ORF1 genotype	Accession No.	5	11	12	14	22	23	25	26	28	32	34	35	36	37
1	GII.P20	EU424333	V	P	L	T	S	N	L	I	T	L	K	G	V	R
	GII.P6	AB039778	•	•	•	•	•	•	•	•	•	•	•	•	•	K
	GII.P7	AB039777	•	•	•	•	•	•	•	•	•	•	•	•	•	•
	GII.P8	AB039780	•	•	•	•	•	•	•	•	•	•	•	•	I	•
2	GII.P1	U07611	I	N	F	S	P	S	F	•	S	I	Q	•	T	Q
	GII.P2	DQ456824	•	•	•	•	•	•	•	•	•	•	•	•	I	T
	GII.P3	KJ194500	V	•	•	T	•	•	•	•	•	•	K	•	•	•
	GII.P4	AB541272	I	S	•	S	•	•	•	•	•	•	Q	•	A	K
	GII.P5	KJ196288	•	•	•	•	•	•	•	•	•	•	S	•	V	T
	GII.P12	AB220922	•	N	•	•	•	•	•	•	•	•	Q	•	A	Q
	GII.P16	KJ196286	•	S	•	•	•	•	•	•	•	•	A	•	I	T
	GII.P17	AB983218	V	N	•	T	•	•	•	•	•	•	K	•	•	•
	GII.P21	KJ196284	I	•	•	S	•	•	•	•	•	•	Q	•	A	Q
	GII.P30	AY134748	•	•	•	•	•	•	•	•	•	•	•	•	T	•
	GII.P31	JX459907	•	•	•	•	•	•	•	V	•	•	•	•	A	K
	GII.P32	MF405169	•	•	•	•	•	•	•	I	•	•	•	•	I	T
	GII.P33	GQ845370	•	•	•	•	•	•	•	V	•	•	•	•	A	Q
	GII.P35	KC576911	•	•	•	•	•	•	•	I	•	•	•	•	•	•
	GII.P37	KJ194507	•	•	•	•	•	•	•	•	•	•	•	•	•	•
	GII.P39	FJ537134	•	•	•	•	•	•	•	•	•	•	•	•	T	•
	GII.P41	JX846924	•	•	•	•	•	•	•	•	•	•	•	•	S	•
	GII.PNA7	MG557653	•	•	•	•	•	•	•	•	•	•	•	•	A	•
3	GII.P24	MG495081	L	•	•	•	S	N	•	•	•	•	•	N	M	S
	GII.P25	MG495083	•	•	•	•	•	•	L	•	•	•	P	•	•	T
	GII.P40	DQ366347	•	•	•	•	•	•	•	•	•	•	•	•	•	•
	GII.PNA5	MG495081	•	•	•	•	•	•	•	•	•	•	•	•	•	A

The squares of negative selection sites are coloured green.

Table S3 (continued). The substitution sites and the negative selection sites of NoV GII strains.

Lineage	ORF1 genotype	Accession No.	39	43	44	45	47	49	51	57	58	62	63	64	66	67
1	GII.P20	EU424333	L	D	I	K	V	I	K	R	F	R	P	I	P	D
	GII.P6	AB039778	•	•	V	•	I	•	•	•	•	K	•	•	•	•
	GII.P7	AB039777	•	E	I	•	**•**	•	•	•	•	R	•	•	•	•
	GII.P8	AB039780	•	D	•	•	V	V	•	•	•	K	•	V	•	•
2	GII.P1	U07611	F	S	•	•	I	I	•	•	L	•	•	I	T	•
	GII.P2	DQ456824	A	P	•	R	V	•	•	•	•	•	•	•	•	•
	GII.P3	KJ194500	•	•	M	N	I	•	•	•	•	•	•	•	P	•
	GII.P4	AB541272	F	•	I	K	**•**	**V**	•	•	**•**	**•**	•	•	T	•
	GII.P5	KJ196288	V	•	V	•	V	I	•	•	F	•	•	•	•	•
	GII.P12	AB220922	F	•	I	•	I	•	•	•	L	**•**	•	•	•	•
	GII.P16	KJ196286	**A**	•	•	•	V	V	•	•	F	•	•	•	P	•
	GII.P17	AB983218	•	•	•	N	I	I	•	•	L	•	•	•	•	•
	GII.P21	KJ196284	F	•	•	K	•	•	•	•	**•**	•	•	•	T	•
	GII.P30	AY134748	•	•	•	•	•	•	•	•	•	•	S	•	•	A
	GII.P31	JX459907	•	•	•	•	•	•	•	•	**•**	•	P	•	•	D
	GII.P32	MF405169	A	•	•	R	•	•	•	•	•	•	•	•	•	•
	GII.P33	GQ845370	F	S	•	K	•	•	•	•	•	•	•	•	•	•
	GII.P35	KC576911	•	P	•	•	•	•	•	•	•	•	•	•	•	•
	GII.P37	KJ194507	•	•	V	•	•	V	•	•	•	•	•	•	•	•
	GII.P39	FJ537134	•	S	I	•	•	I	•	•	•	•	•	•	•	•
	GII.P41	JX846924	•	•	•	•	•	•	•	•	•	•	•	•	•	•
	GII.PNA7	MG557653	•	P	•	•	•	•	•	•	M	•	•	•	•	•
3	GII.P24	MG495081	A	•	•	N	•	•	R	K	•	•	A	•	P	•
	GII.P25	MG495083	•	•	•	S	•	•	•	•	•	•	•	•	•	•
	GII.P40	DQ366347	•	•	•	•	•	V	•	•	•	•	•	•	•	•
	GII.PNA5	MG495081	•	•	•	N	•	I	•	•	•	•	•	•	•	•

The squares of negative selection sites are coloured green.

Table S3 (continued). The substitution sites and the negative selection sites of NoV GII strains.

Lineage	ORF1 genotype	Accession No.	69	71	72	83	84	85	87	90	94	95	99	104	106	108
1	GII.P20	EU424333	T	L	I	C	S	I	V	P	M	I	V	H	S	K
	GII.P6	AB039778	S	•	V	•	•	•	•	•	•	•	•	•	•	•
	GII.P7	AB039777	T	•	•	•	•	•	I	•	•	•	•	•	•	•
	GII.P8	AB039780	S	•	I	•	•	V	•	S	•	•	•	•	•	•
2	GII.P1	U07611	T	M	•	A	T	L	•	P	L	M	A	•	T	•
	GII.P2	DQ456824	S	•	•	•	•	I	•	T	•	•	•	•	•	R
	GII.P3	KJ194500	•	•	•	•	S	•	•	•	•	•	•	•	•	K
	GII.P4	AB541272	T	•	•	V	T	L	•	P	•	•	•	•	•	•
	GII.P5	KJ196288	•	•	•	A	•	I	•	•	•	•	•	•	•	•
	GII.P12	AB220922	•	•	•	V	•	L	•	•	•	•	•	•	•	•
	GII.P16	KJ196286	•	•	•	A	•	V	•	•	•	•	•	•	•	R
	GII.P17	AB983218	S	•	•	V	S	I	•	T	•	•	V	•	•	K
	GII.P21	KJ196284	T	•	•	A	T	L	•	P	•	•	A	•	•	•
	GII.P30	AY134748	•	•	•	V	S	•	•	•	•	•	•	•	•	•
	GII.P31	JX459907	•	•	•	A	T	•	•	•	•	•	•	•	•	•
	GII.P32	MF405169	S	•	•	•	•	I	•	T	•	•	•	•	•	•
	GII.P33	GQ845370	T	•	•	•	•	L	•	P	•	•	•	•	•	R
	GII.P35	KC576911	•	•	•	•	•	•	•	•	•	•	•	•	•	K
	GII.P37	KJ194507	•	•	•	•	S	•	•	•	•	•	•	•	•	•
	GII.P39	FJ537134	•	•	•	•	T	•	•	•	•	•	•	•	•	•
	GII.P41	JX846924	•	•	•	•	•	•	•	•	•	•	•	•	•	•
	GII.PNA7	MG557653	S	•	•	V	•	•	•	•	•	•	•	Q	•	•
3	GII.P24	MG495081	•	•	•	•	•	I	•	T	•	•	•	H	•	•
	GII.P25	MG495083	•	•	•	•	•	•	•	•	•	•	•	•	•	•
	GII.P40	DQ366347	•	•	•	•	•	•	•	•	•	•	•	•	•	•
	GII.PNA5	MG495081	•	•	•	•	•	•	•	•	•	•	•	•	•	•

The squares of negative selection sites are coloured green.

Table S3 (continued). The substitution sites and the negative selection sites of NoV GII strains.

Lineage	ORF1 genotype	Accession No.	112	113	114	125	129	135	144	148	151	152	153	154	174	175	179
1	GII.P20	EU424333	R	T	V	A	N	I	I	G	I	V	V	A	P	D	V
	GII.P6	AB039778	•	•	•	•	•	G	•	•	•	•	•	•	Q	•	•
	GII.P7	AB039777	•	•	•	•	•	•	•	•	•	•	•	•	•	•	•
	GII.P8	AB039780	•	•	•	•	•	•	•	•	•	•	•	•	P	•	•
2	GII.P1	U07611	•	•	•	S	S	T	•	•	Y	•	•	I	N	E	I
	GII.P2	DQ456824	•	•	•	•	•	•	•	•	•	I	•	•	S	•	T
	GII.P3	KJ194500	•	•	•	•	N	•	V	•	F	V	•	•	•	•	•
	GII.P4	AB541272	•	•	•	•	S	•	I	•	Y	•	•	•	•	•	•
	GII.P5	KJ196288	•	M	•	•	G	•	•	•	•	I	•	•	•	•	•
	GII.P12	AB220922	•	T	•	•	S	•	•	•	•	V	•	•	•	•	•
	GII.P16	KJ196286	•	M	•	•	G	•	•	•	•	•	•	•	•	•	•
	GII.P17	AB983218	•	T	•	•	S	•	•	•	F	•	A	•	•	•	•
	GII.P21	KJ196284	•	•	•	•	•	•	•	•	Y	•	V	•	•	•	M
	GII.P30	AY134748	•	•	•	•	•	•	•	•	•	•	•	•	•	•	T
	GII.P31	JX459907	•	•	•	•	•	•	•	•	•	•	•	•	•	•	•
	GII.P32	MF405169	•	•	•	•	•	•	•	•	•	•	•	•	•	•	•
	GII.P33	GQ845370	•	•	•	•	•	•	•	•	•	•	•	•	•	•	V
	GII.P35	KC576911	•	•	•	•	•	•	•	E	•	•	•	•	N	•	T
	GII.P37	KJ194507	•	V	•	•	N	•	•	G	•	I	•	•	S	•	M
	GII.P39	FJ537134	•	T	•	•	S	•	•	•	•	V	•	•	•	•	T
	GII.P41	JX846924	•	•	•	•	•	•	•	•	•	•	•	•	•	•	M
	GII.PNA7	MG557653	•	•	•	•	•	•	•	•	•	I	•	•	•	•	•
3	GII.P24	MG495081	•	M	I	•	N	V	V	•	W	V	•	•	P	D	T
	GII.P25	MG495083	K	•	L	•	•	I	•	•	•	•	•	•	•	•	•
	GII.P40	DQ366347	•	•	•	•	•	•	•	•	•	•	•	•	•	•	•
	GII.PNA5	MG495081	•	•	•	•	•	•	•	•	•	•	•	•	•	•	•

The squares of negative selection sites are coloured green.

Table S4. Parameters for evolutionary rates and Bayesian skyline plot analyses in NoV GII *Pro* regions.

Dataset	Number of strains	Substitution model	Clock model	Tree prior model	Length of MCMC chain	Log parameter
NoV GII	760	GTR+Γ+I	Relaxed Clock Exponential	Coalescent Exponential Population	400,000,000	8,000
GII.P2	10	TPM2+I	Relaxed Clock Exponential	Coalescent Bayesian Skyline	50,000,000	1,000
GII.P4	389	K80+Γ+I	Relaxed Clock Exponential	Coalescent Exponential Population	300,000,000	6,000
			Relaxed Clock Exponential	Coalescent Bayesian Skyline	500,000,000	200,000
GII.P7	39	TPM2+Γ+I	Relaxed Clock Log Normal	Coalescent Exponential Population	250,000,000	5,000
			Relaxed Clock Log Normal	Coalescent Bayesian Skyline	250,000,000	20,000
GII.P12	17	K80+Γ	Relaxed Clock Log Normal	Coalescent Constant Population	50,000,000	1,000
			Relaxed Clock Exponential	Coalescent Bayesian Skyline	50,000,000	1,000
GII.P16	111	TrNef+Γ	Relaxed Clock Exponential	Coalescent Exponential Population	50,000,000	1,000
			Relaxed Clock Exponential	Coalescent Bayesian Skyline	50,000,000	10,000
GII.P17	32	K80+Γ	Relaxed Clock Exponential	Coalescent Exponential Population	300,000,000	6,000
			Relaxed Clock Exponential	Coalescent Bayesian Skyline	50,000,000	2,000
GII.P21	44	TrNef+Γ	Relaxed Clock Exponential	Coalescent Constant Population	50,000,000	1,000
			Relaxed Clock Exponential	Coalescent Bayesian Skyline	50,000,000	5,000
GII.P31	73	K80+Γ	Relaxed Clock Exponential	Coalescent Constant Population	50,000,000	1,000
			Relaxed Clock Exponential	Coalescent Bayesian Skyline	100,000,000	10,000

The parameters for evolutionary rates and BSP are indicated in upper and lower lines, respectively.

The lines of NoV GII and GII.P2 indicated for the parameters of evolutionary rate or BSP, respectively.
